# Multi-omics analysis of organ-specific hormone distribution and molecular regulatory mechanisms in *Cinnamomum burmanni*

**DOI:** 10.3389/fpls.2025.1662457

**Published:** 2025-09-19

**Authors:** Tanhang Zhang, Jun Yao, Qian Zhang, Yanling Cai, Huiming Lian, Minghuai Wang, Jielian Chen, Huihui Zhang, Chen Hou

**Affiliations:** ^1^ Key Laboratory of Saline-alkali Vegetation Ecology Restoration, Ministry of Education, College of Life Sciences, Northeast Forestry University, Harbin, China; ^2^ Guangdong Provincial Key Laboratory of Silviculture, Protection and Utilization, Guangdong Academy of Forestry, Guangdong Academy Of Forestry, GuangZhou, China

**Keywords:** *Cinnamomum burmanni*, plant hormone, metabolome, transcriptome, full-length transcriptome

## Abstract

*Cinnamomum burmanni* serves as a principal arboreal species utilized for the extraction of essential oils, and its foliage and branches contain a wide array of terpenoid compounds. These compounds are extensively utilized in the cosmetic and pharmaceutical sectors. However, the organ-specific distribution of phytohormones and the underlying molecular regulatory mechanisms in *C. burmanni* have not been fully elucidated. Consequently, this study presents the first comprehensive metabolomic, transcriptomic, and full-length transcriptomic analyses aimed at systematically elucidating the organ-specific hormone distribution and molecular regulatory networks within the leaves, stems, and roots of borneol-type *C. burmanni*. The research identified 70 significantly differential hormones, including 32 cytokinin (CTK)-related hormones, 19 auxin-related hormones, and seven gibberellin (GA)-related hormones, uncovering distinct organ-specific patterns: indole-3-acetic acid (IAA) predominantly accumulated in leaves, while GA and CTK were highly expressed in stems. Additionally, 812 differentially expressed genes (DEGs) were identified among different organs, including 50 hormone signaling-related DEGs pinpointed via weighted gene co-expression network analysis (WGCNA). Further investigations indicated that several putative transcription factors (TFs), including ARF, bHLH (PIF3/4), GRAS (DELLA), G2-like (GLK/KAN1/2/HH2O/APL/FT), and ARR-B, may constitute a core regulatory module that mediates hormone-dependent growth, development, and terpenoid biosynthesis. This study establishes the first multi-omics-driven hormonal interaction network framework for the molecular breeding of *C. burmanni* while developing a gene editing target atlas to elucidate synergistic regulatory mechanisms underlying medicinal secondary metabolite biosynthesis.

## 1 Introduction

*Cinnamomum burmanni* is an essential oil tree species belonging to the Lauraceae family (Singh et al., 2021). It has aromatic properties (Ma et al., 2021; Liu et al., 2023; Zhang et al., 2024), and its leaves and stems contain abundant terpenoids (Yang et al., 2020; Li et al., 2022; Ma et al., 2022; Huang et al., 2024), which are widely utilized in the pharmaceuticals and cosmetics industries (Al-Dhubiab, 2012). However, we encountered difficulties in the root formation of *C. burmanni* when using the cutting and tissue culture propagation methods for *Cinnamomum camphora*, which likewise belongs to the Lauraceae family (Huang et al., 1998; Babu et al., 2003; Asare et al., 2014). Several phytohormones have been reported to promote the formation of adventitious roots and buds of Lauraceae plants. Luo et al. (2024) reported that auxin, ethylene, and the signaling pathways of plant wound play crucial roles in the process of adventitious root growth in *Cinnamomum parthenoxylon* cuttings. Luo et al. (2025) reported that zeatin riboside (ZR), abscisic acid (ABA), gibberellin (GA), and the [indole-3-acetic acid (IAA) + GA + ZR]/ABA ratio play vital roles in the formation of adventitious buds and roots of *C. parthenoxylon*. Hence, we performed multi-omics analysis to identify genes that are involved in hormone signal transduction in *C. burmanni*, which offer the foundation for further investigations of adventitious root induction and growth regulation.

Phytohormones, including auxin, cytokinins (CTKs), GAs, and ABA, are key signaling compounds biosynthesized by plants (Waadt et al., 2022). They can be translocated at extremely low concentrations and modulate the differential processes of development, growth, and response to stress in plants (Anfang and Shani, 2021; Zhang et al., 2023). Auxin, GA, and CTK, which are three crucial plant hormones, influence plant developmental processes and growth (Powell and Heyl, 2023). IAA, which is the most prevalent bioactive form (Friml, 2022), participates in the processes of growth and development in plants, including organ development, phototropism, directional root growth, and stress response (Anfang and Shani, 2021). In Moso bamboo (*Phyllostachys edulis*), the overexpression of *PedARF23* activates signal transduction, which enhances auxin biosynthesis (Hu et al., 2025). Conversely, the mutation of *ARFTF17* reduces IAA content in the pericarp of *Zea mays* seeds and inhibits auxin biosynthesis, leading to the phenotype of flint-like seeds (Wang et al., 2024). GAs participate in stem elongation, leaf senescence, floral development, and seed germination (Guo et al., 2022; Zhang et al., 2023), but only gibberellin A1 (GA1), gibberellin A3 (GA3), gibberellin A4 (GA4), and gibberellin A7 (GA7) display biological activity (Rizza and Jones, 2019; Wu et al., 2021; Zhou et al., 2022). In *Solanum lycopersicum*, the overexpression of *DoDELLA1* significantly suppresses endogenous GA_1_ and GA_3_ levels while downregulating GA metabolic genes, ultimately leading to dwarfism and delaying flowering in transgenic tobacco (Zhou et al., 2022). CTKs orchestrate key physiological processes in plants, including shoot meristem maintenance, cambial activity, cell division, cell differentiation, vascular development, secondary growth, organ development, and stress adaptation (Abdel Latef et al., 2021; Powell and Heyl, 2023; Svolacchia and Sabatini, 2023). In *Lolium perenne*, *LpARR10* may transactivate the *SOS1* and *SOS3* genes, which are two salt overly sensitive (SOS) genes, via CTK signal transduction to enhance salinity tolerance (Yang et al., 2024).

**Figure 1:**
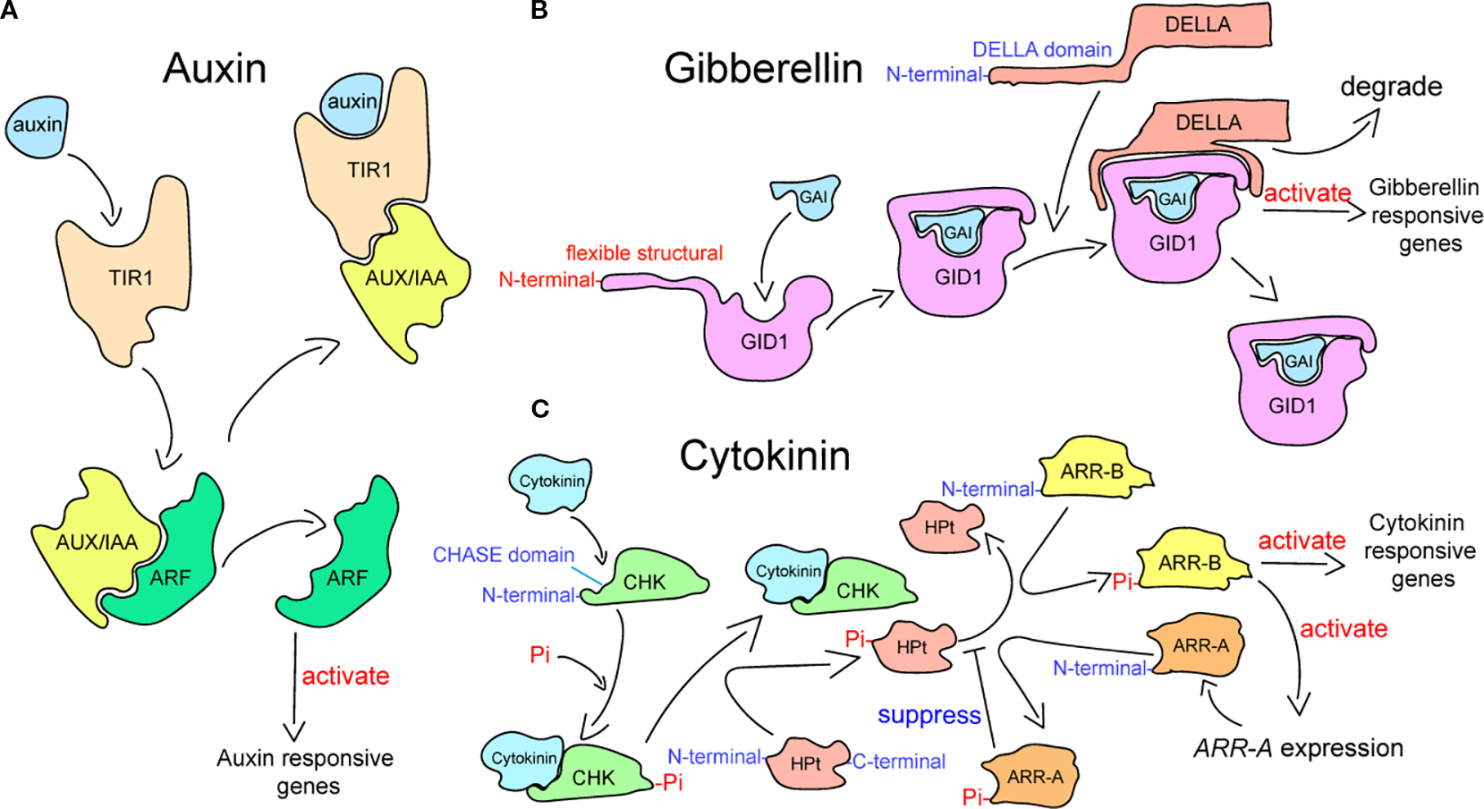
Plant hormone signal transduction processes in plants. **(A)** Auxin signaling transduction pathway, which has been adapted from Yu et al. (2022). **(B)** Gibberellin signaling transduction pathway, which has been adapted from Murase et al. (2008). **(C)** Cytokinin signaling transduction pathway, which has been adapted from Tan et al. (2019).

Plant hormone signaling pathways interact antagonistically or synergistically to determine the downstream signaling events that are activated by hormones (Berens et al., 2017). The crosstalk of hormone signaling is formed by the interactions of plant hormones, mediating the immune responses and growth processes under abiotic stress (Pieterse et al., 2009; Spaepen and Vanderleyden, 2011; Shigenaga and Argueso, 2016). The transduction of auxin signaling in plants is governed by three key signaling constituents: transcription factors auxin response factors (ARFs), transcriptional repressor auxin/IAA (Aux/IAA), and receptor Transport Inhibitor Response 1/Auxin Signaling F-box Protein (TIR1/AFB) (**Figure 1A**) (Salehin et al., 2015; Leyser, 2018; Matthes et al., 2019; Niemeyer et al., 2020). Auxin acts as the key agent determining the proteins that bind to Aux/IAA proteins in this process (Yu et al., 2022). Aux/IAAs combine with ARFs at low auxin concentrations, repressing the transcriptional activity of downstream auxin-responsive genes. When exhibiting a certain concentration, auxin leads to the establishment of the TIR1–auxin–Aux/IAA complex after entering the pocket of TIR1 (Tan et al., 2007; Calderón Villalobos et al., 2012; Wang and Estelle, 2014). The complex triggers the degradation of Aux/IAA via the ubiquitination of the 26S proteasome, which releases the ARF to stimulate the expression of auxin-responsive genes (Weijers and Wagner, 2016). The signal transduction of GA is related to the degradation of DELLA transcription regulators, which is initiated through the process by which bioactive GAs are recognized by the GA receptor that is encoded by *GIBBERELLIN INSENSITIVE DWARF1* (*GID1*) (**Figure 1B**) (Blázquez et al., 2020). The GA receptor is present in the nucleus and cytoplasm (Ueguchi-Tanaka et al., 2005; Shimada et al., 2008), which features a flexible structural motif in N-terminal extension (N-ex) and a GA-binding pocket in the C-terminal domain (Ueguchi-Tanaka and Matsuoka, 2010; Livne and Weiss, 2014). Upon binding to the C-terminal pocket, bioactive GAs induce the folding of the N-ex over this pocket, thereby exposing a surface on GID1 that enables binding to the DELLAs (Murase et al., 2008). The DELLA subfamily belongs to the GRAS family (Vera-Sirera et al., 2016; Li et al., 2024), which possesses a DELLA domain that is recognized by GID1 and required for GA-induced degradation in the N-terminal (Ueguchi-Tanaka et al., 2007; Zhou et al., 2024b). After bioactive GAs are recognized by GID1, the complex binds to DELLA proteins to facilitate their degradation (Claeys et al., 2014; Hernández-García et al., 2021). CTK signaling is perceived and transduced by the two-component system (TCS) (Zameer et al., 2021), composed of two key constituents: a response regulator that mediates downstream interactions upon phosphorylation-dependent activation by the receptor and a receptor kinase that undergoes autophosphorylation after perceiving the signal (**Figure 1C**) (Hwang and Sheen, 2001; Gruhn and Heyl, 2013; Kieber and Schaller, 2018; Wang et al., 2025a). Cytokinin is sensed by hybrid histidine kinase receptors (CHKs), which contain the cyclase/histidine kinase-associated sensing extracellular (CHASE) domain at the endoplasmic reticulum (ER) membrane and plasma membrane (PM) (Romanov et al., 2018; Antoniadi et al., 2020; Kubiasová et al., 2020; Powell and Heyl, 2023). Next, CHKs relay the signal to the histidine-containing phosphotransfer (HPt) proteins by autophosphorylating (Hutchison et al., 2006). Once the phosphorylated HPts move into the nucleus from the cytoplasm, they transfer the phosphorylation signal, leading to the phosphorylation of the response regulators (Punwani et al., 2010). In plants, CTK signaling involves two distinct types of response regulators: B-type response regulators (ARR-Bs) and A-type response regulators (ARR-As) (Lai et al., 2021). ARR-Bs, which are MYB transcription factors, are phosphorylated by phosphorylated HPts, activating the expression of genes that respond to a cytokinin signal, including ARR-As (Powell and Heyl, 2023). In contrast, ARR-As, which compete with ARR-Bs for the phosphorylation signal of phosphorylated HPts, negatively regulate cytokinin signal transduction (Hwang and Sheen, 2001).

Transcription factors (TFs), which are crucial protein factors, serve a pivotal function in the hormone signal transduction pathways by modulating the transcriptional regulation level of target genes to regulate the growth and development of plants and response (Yoon et al., 2020; Shrestha et al., 2021; Donovan and Larson, 2022; Li et al., 2023b; Thilakarathne et al., 2025), including ARF, bHLH, GRAS, G2-like, and ARR-B (Chen et al., 2020; Wang et al., 2020a, b; Li et al., 2021; He et al., 2022). The transcriptional activity of auxin-responsive genes is activated by ARF proteins and inhibited by Aux/IAA proteins by binding to ARFs (Yang et al., 2022; Li et al., 2023a). In *Bambusa emeiensis*, *PedARF23* regulates lignin biosynthesis by activating the signaling pathway of auxin to promote the biosynthesis of auxin (Hu et al., 2025). In *Dendrobium officinale*, Aux/IAA proteins (DoIAA10, 13) and ARF proteins (DoARF2, 17) coordinately regulate auxin signal transduction to control floral development (Si et al., 2023). The DELLA proteins, which are positively regulated by GA biosynthesis (Ohama et al., 2025), predominantly inhibit GA signal transduction (Van De Velde et al., 2017). In *Arabidopsis thaliana*, bioactive GAs bind to GID1, facilitating the degradation of DELLA proteins to regulate *GIBBERELLIN INSENSITIVE* (*GAI*) (Murase et al., 2008). In wheat (*Triticum aestivum*), the photoreceptor CRY1 competitively inhibits DELLA proteins to suppress GA signal transduction, enhancing its inhibitory effect on plant growth (Yan et al., 2021). The CTK signaling pathway involves ARR-Bs and ARR-As (Lai et al., 2021; Powell and Heyl, 2023). ARR-Bs positively regulate the CTK signaling pathway (Zhou et al., 2024a), and ARR-As negatively regulate the CTK signaling pathway (Tan et al., 2019; Lu et al., 2023). In *Medicago truncatula*, the CTK signaling pathway mechanism relies on the TCS. After perceiving the phosphorylated signal from phosphorylated HPts, ARR-Bs are phosphorylated to positively regulate the transcriptional level of cytokinin response genes, while ARR-As compete for the phosphorylation signal that generates negative feedback in cytokinin signal transduction (Tan et al., 2019).

Notably, the processes of growth and development in Lauraceae are influenced by diverse phytohormone signal transductions (Guo et al., 2024a). In *Cinnamomum bodinieri*, Yu et al. (2025) reported that IAA, ABA, ZR, and GA play central roles in adventitious root (AR) formation, with IAA as the primary regulator. They identified 28 differentially expressed genes (DEGs) of the hormone signaling pathway (including *CYP94B3* and *NAC82*) and confirmed that transcription factors (such as AUX1, GH3, ZFP, NAC, ARR-A, and ARR-B) participate in this process. In ancient *C. camphora* leaves, Liu et al. (2024) found that IAA, iP, and iPR positively influence regeneration after cutting. They also identified 57 DEGs of the hormone signaling pathway and revealed that transcription factors, including AUX1, GH3, SAUR, bAHP, CYCD3, SIMKK, EBF1/2, ARR-B, BKI1, BSK, JAZ, MYC2, TGA, and PR-1, play roles in hormone signal transduction. Currently, previous research has primarily focused on terpenoid biosynthesis in Lauraceae plants (Luo et al., 2022; Ma et al., 2022; Ju et al., 2023), whereas studies on hormone signaling pathways remain limited, with no reports specifically on *C. burmanni*. Furthermore, the distribution patterns and regulatory mechanisms of hormones across different organs remain largely unexplored in Lauraceae. Therefore, we integrated metabolomic, transcriptomic, and full-length transcriptomic analyses to systematically identify DEGs that engage in hormone signaling and differentially expressed hormones in differential plant organs of borneol-type *C. burmanni*. Furthermore, the functions of key genes in association with potential TFs were predicted. This study not only fills the knowledge gap in hormone distribution, signaling, and molecular regulation across multiple organs in Lauraceae but also provides a theoretical framework for future breeding strategies to improve *C. burmanni* cultivars and enhance stress tolerance.

## 2 Materials and methods

### 2.1 Plant materials

Multiple organ plant materials were obtained from a 3-year-old borneol-type *C. burmanni*, which was cultivated at the Guangdong Academy of Forestry Sciences (113°37′37.36″N, 23°19′42.08″E, Guangzhou, China). The mother plant originated from a *C. burmanni* individual with a highly borneol-expressing chemotype, which was selectively bred by our research team in Tianlu Lake Forest Park (113°25′54″N, 23°15′33.4″E, Guangzhou, China). Among the propagated cuttings, this individual was one of the few successfully rooted branches and exhibited the most vigorous growth when sampled. The cuttings were cultivated in a potting mix that was composed of 90% peat soil and 10% perlite (v/v). A rooting powder (including naphthaleneacetic acid, indolebutyric acid, and talcum powder) that was developed by the research team was applied. The climatic conditions included the following: the annual mean temperature was 29.3°C, the mean relative humidity was 77%, extreme highs were 39°C, extreme lows were 3°C, annual precipitation was 1,353.5 mm, and the frost-free period was ≥340 days. The Resource Management Department of Longdong Forest Farm granted permission for the collection of plant materials. The researchers performed hormone metabolomic and transcriptomic analyses on the roots, stems, and leaves, with three biological replicates prepared per tissue. Additionally, nine samples (roots, stems, and leaves) were pooled for full-length transcriptome sequencing. Upon collection, all specimens were swiftly frozen with liquid nitrogen and kept at −80°C.

### 2.2 Metabolomic analysis

Metabolite profiling was performed using liquid chromatography–tandem mass spectrometry (LC-MS/MS), according to methods in Chen et al. (2013) with modifications (China). Nine samples were frozen, dried using a vacuum freeze-dryer (Scientz-100F, Ningbo Scientz Biotechnology Co., Ltd., Ningbo, China), and pulverized into a fine powder using a grinding machine with zirconium oxide beads (30Hz, 1.5min). Powder (100 mg) was weighed and sequentially added with 1 mL methanol/water/formic acid (15:4:1, v/v/v) and 10 μL internal standard (100 ng/mL). After vortexing (10min) and centrifugation (12,000 rpm/min, 5min, 4°C), supernatants were concentrated, redissolved in 80% methanol (100 μL), and filtered using a 0.22-μm membrane filter (SCAA-104, 0.22-μm pore size; ANPEL, http://www.anpel.com.cn/) for analysis.

Chromatographic separation was achieved using a Waters ACQUITY UPLC HSS T3 C18 column (1.8 µm, 100 × 2.1mm) with mobile phases A (0.04% acetic acid in water) and B (0.04% acetic acid in acetonitrile) (Niu et al., 2014). The elution protocol was as follows: 0min, A:B = 95:5 (V:V); 1min, A:B = 95:5 (V:V); 8min, A:B = 5:95 (V:V); 9min, A:B = 5:95 (V:V); 9.1min, A:B = 95:5 (V:V); and 12min, A:B = 95:5 (V:V) (flow rate, 0.35 mL/min; injection volume, 2 μL; column temperature, 40°C). MS detection employed a QTRAP^®^6500+LC-MS/MS system (https://sciex.com.cn/) in multiple reaction monitoring (MRM) mode with an ESI Turbo ion spray interface (voltage, ± 5500/4500V; temperature, 550°C; curtain gas, 35psi) (Niu et al., 2014). Data were acquired using Analyst 1.6.3 (AB Sciex, Framingham, MA, USA) and analyzed using MultiQuant 3.0.3. According to the Metware Database, a triple quadrupole mass spectrometer was employed for qualitative analysis of the data, while quantitative analysis of the data was performed using the MRM mode in a triple quadrupole mass spectrometer (MS2 tolerance, 20 ppm; Retention Time (RT) error, 0.2min).

Hormone standard solutions (0.01, 0.05, 0.1, 0.5, 1, 5, 10, 50, 100, 200, and 500 ng/mL) were prepared, with l-tryptophan (TRP) and salicylic acid 2-*O*-β-glucoside (SAG) standard curves ranging from 0.2 to 10,000 ng/mL (20 times dilution). The mass spectral peak intensities of the quantitative signals of hormone standards were recorded at each concentration, and standard curves were generated (**Supplementary Table 1**).

To enhance the normal distribution, metabolite data were log2-transformed for normalization. The prcomp statistical function from the R software (version 4.2.2) was employed for principal component analysis (PCA) (base package, version 3.5.1) of metabolites across nine samples. Subsequently, hierarchical cluster analysis (HCA) was conducted, and the ComplexHeatmap package (Gu et al., 2016) from the R software was utilized to construct heatmaps. In addition, the R software package MetaboAnalystR (https://gitcode.com/gh_mirrors/me/MetaboAnalystR) was employed to construct an orthogonal partial least squares discriminant analysis (OPLS-DA) model to select significantly differential hormones among distinct samples, with Fold Change ≥ 2 and Fold Change ≤ 0.5 as the selection thresholds. The Kyoto Encyclopedia of Genes and Genomes (KEGG) database (https://www.genome.jp/kegg) was employed for the enrichment analysis of the significantly differential hormones. The absolute hormone levels were calculated based on the peak area ratios with a standard curve using the linear equation. Data visualization was conducted using GraphPad Prism v6.01 (https://www.graphpad.com).

### 2.3 Full-length transcript sequencing and analysis

The extraction of the total RNA of each sample was performed using the FastPure^®^ Universal Plant Total RNA Isolation Kit. A 1% agarose gel electrophoresis (180V) was employed to assess RNA integrity and potential DNA contamination. The OD260/230 and OD260/280 values were measured using the NanoPhotometer spectrophotometer (IMPLEN, Westlake Village, CA, USA) to determine the purity of total RNA. Next, precise determination of the RNA concentration was conducted using the Qubit 2.0 Fluorometer (Life Technologies, Carlsbad, CA, USA), and evaluation of RNA integrity was conducted employing the Agilent 2100 Bioanalyzer (Agilent Technologies, Santa Clara, CA, USA).

The Oligo(dT) magnetic bead approach was applied to construct a full-length transcriptome library in this research. The mRNAs that contained polyA were enriched with Oligo(dT) magnetic beads. Subsequently, a SMARTer PCR cDNA synthesis kit was utilized to transform mRNA to cDNA, which was further enriched for synthesis. The optimal conditions for downstream PCR were determined by optimizing experimental procedures. Large-scale PCR was performed using Oligo(dT) bead-selected fragments to construct a SMRTbell library. The cDNA of full-length was linked to sequencing adapters. Subsequent to the annealing process, the SMRTbell template was utilized as sequencing primers bound to the polymerase. The PacBio Sequel platform was subsequently employed to conduct a 10-hour sequencing run, which utilized P6-C4 chemistry. The raw data of the full-length sequencing have been deposited in the NCBI Sequence Read Archive (SRA) with project number PRJNA1287254.

The initial sequencing data were filtered using the PacBio official software package SMRTlink v8.0 (https://www.pacb.com/support/software-downloads/) with the following parameters: the minimum length was configured as 50 bp, the maximum length was adjusted to 15,000 bp, and the minimum number of full passes was set to one. After correcting the subreads, Circular Consensus Sequences (CCSs) were obtained. CCSs were categorized as Full-Length (FL) sequences and non-Full-Length (nFL) sequences based on the presence of 5′, 3′ primers and polyA tail signals. Within the FL sequences, the Full-Length Non-Chimeric (FLNC) sequences corresponding to the same transcript were analyzed using a hierarchical clustering algorithm [hierarchical nlog(n)] to facilitate the removal of redundant sequences and the derivation of cluster consensus sequences. Subsequently, cluster consensus sequences were polished and yielded consensus sequences with high fidelity. Consensus sequences with high accuracy were utilized via the Arrow (Chin et al., 2013) for the generation of polished consensus sequences for subsequent analyses.

The polished consensus sequences were employed using the GMAP v2017-06-20 (Genomic Mapping and Alignment Program) software to align to the reference genome. The structurally annotated transcripts were subsequently employed for functional annotation using five primary databases. Fictional annotations of these transcripts were performed against KEGG, NR (Non-Redundant Protein Database), and Swiss-Prot (a manually annotated and reviewed protein sequence database) using Diamond blastx v0.8.36 (https://github.com/bbuchfink/diamond). An annotation of Pfam (Protein Family Analysis and Modeling) was performed utilizing Hmmscan v3.1b2 (http://hmmer.org/download.html). Functional annotation of Gene Ontology (GO) was performed by applying NR annotation data and the Blast2GO software (Wu and Watanabe, 2005). Finally, the number of successfully annotated transcripts was statistically analyzed.

The TAPIS (Conesa et al., 2005) software was applied to classify and characterize the full-length transcripts of *C. burmanni*. After aligning to the polished consensus reference genome, the high-quality isoforms were obtained by further correction, clustering, and redundancy removal. Subsequently, transcript characteristics, novel gene and novel transcript identification, novel gene database annotation, and transcription factor analysis and prediction were conducted. The prediction of plant transcription factors was achieved using iTAK (https://github.com/kentnf/iTAK/) (Abdel-Ghany et al., 2016). Coding potential prediction of PacBio sequencing data was carried out using CNCI (Sun et al., 2013), PLEK (Li et al., 2014), the CPC software (Kong et al., 2007), and the Pfam database (Finn et al., 2016).

### 2.4 Transcriptome sequencing and analysis

The integrity and potential DNA contamination of the RNA sample were assessed employing 1% agarose gel electrophoresis (180V). Subsequently, the NanoPhotometer spectrophotometer (IMPLEN, Westlake Village, CA, USA) was employed to ascertain the OD260/230 and OD260/280 ratios to assess the purity of the RNA. Accurate quantification of RNA concentration was performed using the Qubit 2.0 Fluorometer (Life Technologies, Carlsbad, CA, USA), and precise quantification of RNA integrity was performed using the Agilent 2100 Bioanalyzer (Agilent Technologies, Santa Clara, CA, USA).

In this study, 1 μg of total RNA was utilized as the primary sample to construct the sequencing library of transcriptome utilizing the NEBNext^®^ Ultra™ RNA Library Prep Kit (Illumina). Before library construction, the Qubit 2.0 Fluorometer was used to carry out initial quantification and then dilute the library to 1.5 ng/μL. The effective concentration of the library was precisely quantified via qRT-PCR. Upon passing quality control, the Illumina sequencing pipeline was initiated. The raw data of the RNA sequencing have been deposited in the NCBI SRA with project number PRJNA1286930.

To generate clean reads for further analysis, raw sequencing data were treated to filter out sequences of adapters and reads of poor quality using Fastp v0.19.3 (Chen et al., 2018). After downloading the reference genome and its annotation files, the index was generated using the software HISAT v2.1.0 (Kim et al., 2015). Subsequently, the clean reads were aligned against the reference genome. Fragments Per Kilobase per Million mapped reads (FPKM) was used to evaluate the expression level of genes. DESeq2 v1.22.1 and edgeR v1.22.1 were utilized to assess differentially expressed genes among the sample groups. The Benjamini and Hochberg procedure was executed to correct for multiple hypothesis testing by adjusting the probability values (p-values) to yield the false discovery rate (FDR). The threshold for DEGs was determined in accordance with the following criteria: |log2(fold change)| ≥ 1 and q-value < 0.05.

The numbers of DEGs were counted for pairwise comparisons among roots, stems, and leaves as the three groups. The NR, GO, KEGG, Swiss-Prot, and Pfam databases were utilized to annotate all DEGs. Next, the DAVID database (https://david.ncifcrf.gov/home.jsp) was employed for the functional enrichment analysis of GO and KEGG pathways of all DEGs.

To forecast putative TFs that connected with DEGs, which were relevant to hormone signal transduction within three organs in *C. burmanni*, the protein sequences of these DEGs were loaded into PlantTFDB v5.0 (https://planttfdb.gao-lab.org/prediction.php). This database identifies TFs that were based on input sequences, family classification rules, and predefined thresholds (Tian et al., 2020).

Based on transcriptomic data, the *cor*() function within the stats package of the R software (version 4.2.2) was utilized to predict Pearson’s product-moment correlation coefficients (PCCs) between TFs and DEGs (Bai et al., 2022). The correlations with statistical significance (p < 0.05) and absolute values > 0.8 were retained. Co-expression networks were visualized using Cytoscape (version 3.9.1) (Kohl et al., 2011). This study details the comprehensive bioinformatics workflow, as depicted in **Supplementary Figure 1**, which encompasses analyses of full-length transcriptomics, transcriptomics, and metabolomics.

### 2.5 Weighted gene co-expression network analysis

The 10 most abundant hormones, including GA1, GA3, gibberellin A20 (GA20), indole-3-acetyl-l-valine methyl ester (IAA-Val-Me), *N* ^6^-isopentenyl-adenine-9-glucoside (iP9G), *meta*-topolin-9-glucoside (mT9G), *ortho*-topolin-9-glucoside (oT9G), 2-oxindole-3-acetic acid (OxIAA), 4-[[(9-beta-d-glucopyranosyl-9H-purin-6-yl)amino]methyl]phenol (pT9G), and TRP, of different organs were selected for weighted gene co-expression network analysis (WGCNA) using the WGCNA 1.71 package from the R software (version 4.2.2), with FPKM values (R^2^ > 0.85) set at an appropriate threshold (Wang et al., 2022). A hierarchical clustering tree was generated based on the correlation of intergenic expression levels between gene expression and the abundance of hormones. Dynamic tree cutting analysis was utilized to segregate genes exhibiting significant association with the 10 selected hormones into discrete modules, followed by the amalgamation of analogous modules.

### 2.6 qRT-PCR analysis

The investigation utilized the FastPure^®^ Universal Plant Total RNA Isolation Kit for the extraction of RNA and employed the HiScript III RT SuperMix for qPCR (+gDNA wiper) for the reverse transcription process. A total of 12 differentially expressed genes associated with auxin, gibberellin, and cytokinin were randomly chosen and confirmed through qRT-PCR experiments. Root, stem, and leaf samples were collected, and triplicate technical and biological replicates were conducted. The qRT-PCR assay used Actin7 as the internal standard gene. The primer sequences were designed based on the study by Shi et al. (2024). A specific primer was designed using the PrimerQuest™ Tool (https://sg.idtdna.com/pages/products/qpcr-and-pcr). The detailed sequences of the primer are presented in **Supplementary Table 2**. The reaction condition was set as follows: 10 µL 2× ChamQ Universal SYBR qPCR Master Mix, 1.0 µL cDNA, 1.0 µL Primer1 (10 µM), 1.0 µL Primer2 (10 µM), and 8.2 µL ddH_2_O. The procedures of qRT-PCR cycling were configured in the following manner: pre-denaturation at 95°C for 30 seconds, denaturation at 95°C for 10 seconds, and annealing at 60°C for 30 seconds, with 40 cycles. The comparative level of DEG expression was quantified through the 2^−ΔΔCT^ approach (Livak and Schmittgen, 2001).

## 3 Results

### 3.1 Screening of significantly differential hormones in *C. burmanni* by metabolomic analysis

**Figure 2:**
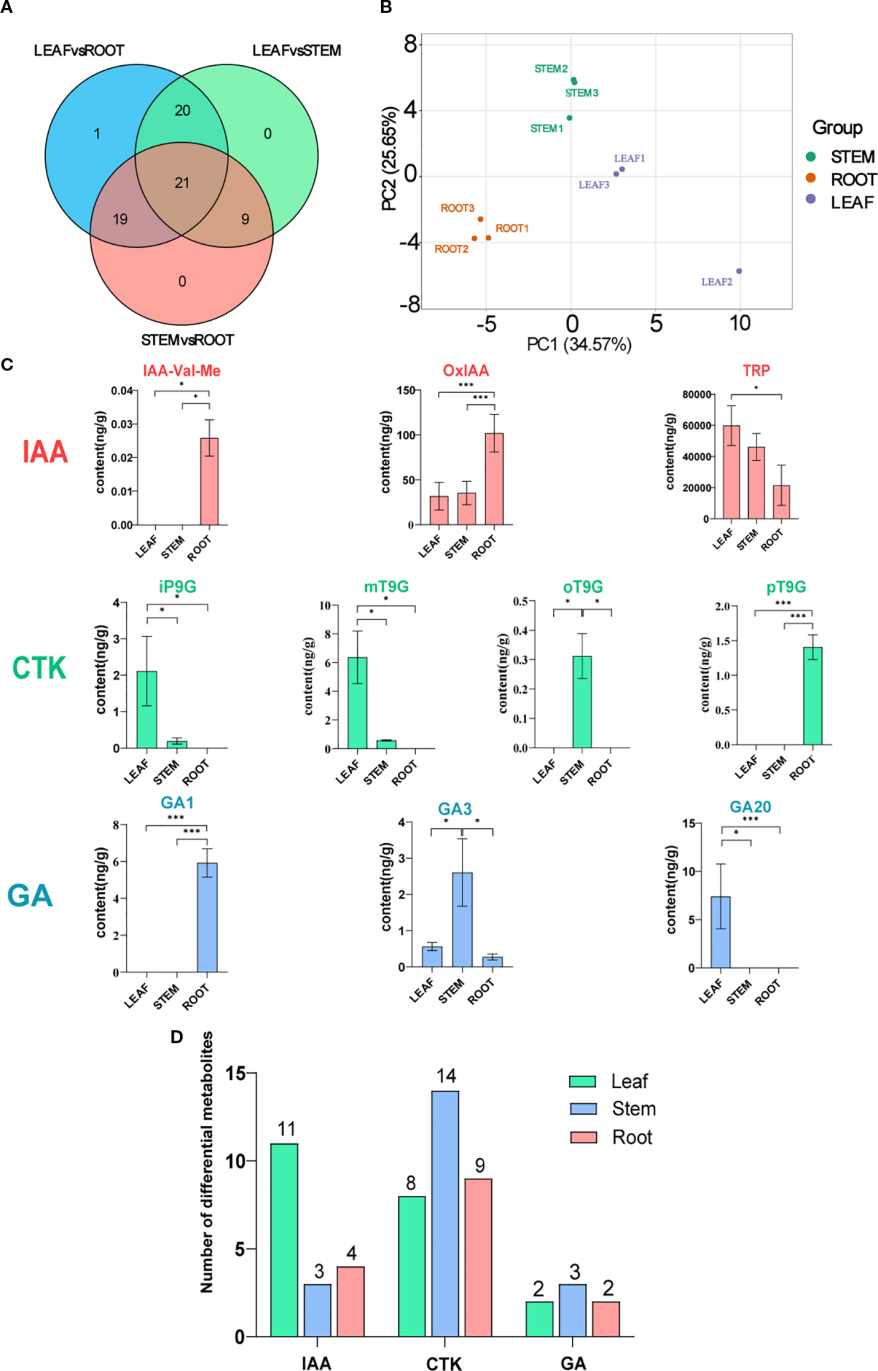
Metabolomic analysis of *Cinnamomum burmanni*. **(A)** Venn diagram illustrating the comparative analysis of differential hormones in distinct tissues of *C*. *burmanni*. **(B)** Principal component analysis (PCA) of metabolomic features from three organs. **(C)** Contents and proportions of 10 highly expressed phytohormones (symbols * and *** indicate p < 0.05 and p < 0.01 quantified through one-way ANOVA as well as t-test, respectively). Salmon color represents indole-3-acetic acid (IAA) hormones, spring green denotes cytokinin (CTK) hormones, and sky blue corresponds to gibberellin (GA) hormones. **(D)** Histogram of differential metabolites across various tissues of *C*. *burmanni*.

Metabolomic analysis was conducted to comprehensively identify the significantly differential hormones present in the root, stem, and leaf tissues of *C. burmanni*. Differential hormones were observed in the roots, stems, and leaves based on the PCA (**Figure 2B**). In our investigation, 72 differential hormones were identified (**Supplementary Table 3**), including 32 CTK hormones (44.44%), 19 auxin hormones (26.38%), seven GA hormones (9.7%), nine jasmonic acid (JA) hormones (12.5%), two salicylic acid (SA) hormones (2.8%), two ABA hormones (2.8%), and one ethylene (ETH) hormone (1.4%). Hormones exhibiting a Fold Change of ≥2 or ≤0.5 were deemed to be significantly differentially expressed, resulting in the identification of a total of 70 such hormones. In this study, auxin hormones exhibited significant enrichment within the LEAF category, with a total of 11 instances, while cytokinin hormones were predominantly found in the STEM category, with 14 instances. Additionally, gibberellin hormones were significantly enriched in STEM, with a count of three (refer to **Figure 2D**). Notably, differential metabolites tryptamine (TRA) and *trans*-zeatin riboside (tZR) were not categorized as significantly differential hormones, as indicated in **Supplementary Table 4**. Venn diagram analysis of significantly differential hormones enriched in the three differential organs showed that 21 significantly differential hormones were commonly enriched across all three tissues (**Figure 2A**), including 10 CTK hormones [6-benzyladenine (BAP), 6-benzyladenosine (BAPR), *cis*-zeatin (cZ), *cis*-zeatin-*O*-glucoside riboside (cZROG), *N* ^6^-isopentenyladenine (IP), kinetin (K), mT9G, oT9G, pT9G, and *trans*-zeatin-*O*-glucoside (tZOG)], five IAA hormones [IAA, indole-3-acetyl-l-tryptophan (IAA-Trp), 3-indoleacetamide (IAM), 3-indolebutyric acid (IBA), and indole-3-carboxaldehyde (ICAld)], two GA hormones [GA3 and gibberellin A24 (GA24)], two JA hormones [JA and 3-oxo-2-(2-(*Z*)-pentenyl) (OPC-4)], and two SA hormones (SA and SAG). Notably, 3-indoleacetonitrile (IAN) was a unique, significantly differential hormone in the LEAFvsROOT group.

The metabolic pathways enriched by significantly differential metabolites in the three organs were revealed through KEGG enrichment analysis. All groups exhibited enrichment in the following hormone-related pathways: “alpha-Linolenic acid metabolism” (ko00592), “Diterpenoid biosynthesis” (ko00904), “Plant hormone signal transduction” (ko04075), “Tryptophan metabolism” (ko00380), and “Zeatin biosynthesis” (ko00908). However, the pathways “Carotenoid biosynthesis” (ko00906) and “Phenylalanine, tyrosine and tryptophan biosynthesis” (ko00400) were exclusively accumulated in ROOTvsLEAF and ROOTvsSTEM (**Supplementary Figures 2A–C**). To further investigate the variations in hormone content across differential organs, 10 hormones that were relatively highly expressed in three distinct organs were selected for comparative analysis, including GA1, GA3, GA20, IAA-Val-Me, iP9G, mT9G, oT9G, OxIAA, pT9G, and TRP. The results demonstrated that LEAF contained higher levels of GA20, iP9G, mT9G, and TRP compared to other organs. STEM exhibited higher levels of GA1, IAA-Val-Me, OxIAA, and pT9G, while ROOT showed higher levels of GA3 and oT9G (**Figures 2C, D**).

### 3.2 Construction of the full-length transcriptome of *C. burmanni*

**Figure 3:**
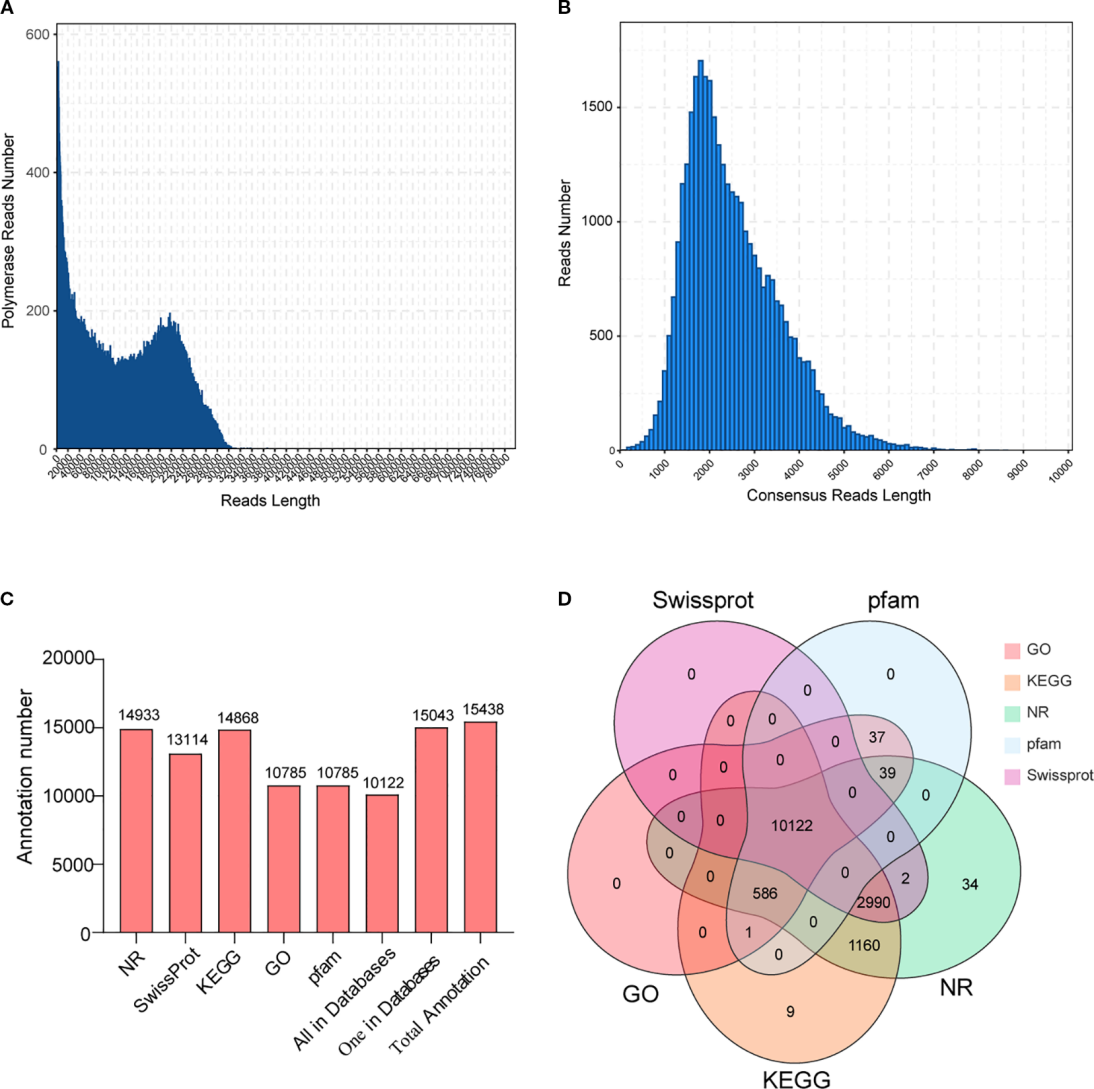
Full-length transcriptome analysis of *Cinnamomum burmanni* roots, stems, and leaves. **(A)** The length distribution of polymerase reads. **(B)** The length distribution of Circular Consensus Sequence (CCS) length. **(C)** Functional annotation of structurally annotated transcripts in NR, Gene Ontology (GO), Kyoto Encyclopedia of Genes and Genomes (KEGG), Pfam, and Swiss-Prot databases. **(D)** Venn diagram of annotation results for full-length transcripts in NR, GO, KEGG, Pfam, and Swiss-Prot databases.

This investigation performed full-length transcriptome analysis to obtain high-quality and as complete as possible transcript sequences in *C. burmanni*. After sequencing, 43.79-GB raw data and 384,075 polymerase reads were obtained, with a polymerase read N50 of 2,931 bp. The distribution of polymerase read lengths is demonstrated in **Figure 3A**. After filtering raw data, 18,738,519 subread sequences were obtained. Upon self-alignment correction of each subread sequence, 354,555 CCSs were acquired. The length distribution of CCSs matched the expected pattern, as illustrated in **Supplementary Figure S3A**. According to the 5′, 3′ primers and tail signals of polyA, the CCSs were categorized into FL and nFL sequences. A total of 316,567 FL sequences and 37,988 nFL sequences were obtained.

Among the FL sequences, 315,605 FLNC and 962 Full-Length Chimeric (FLC) sequences were separated. The distribution of FLNC sequence lengths is depicted in **Supplementary Figure S3B**. First, after clustering and deduplicating, FLNC sequences were utilized to obtain consensus sequences. Subsequently, consensus sequences were adjusted using the Arrow software; 34,245 polished consensus sequences were obtained, and the distribution of polished consensus sequence lengths is displayed in **Figure 3B**, which demonstrates that the majority of these sequences were approximately 2,000 bp in length. The Genomic Mapping and Alignment Program (GMAP) was employed to align between polished consensus sequences and the reference genome, resulting in 30,027 (87.68%) successfully mapped consensus sequences (total mapped).

Among the 15,483 structurally annotated transcripts, 15,043 (97.16%) had annotations no fewer than one within the NR, GO, KEGG, Pfam, or Swiss-Prot database (**Supplementary Table 5**). As illustrated in **Figure 3C**, the annotated transcripts of the highest quantity are in the NR database, and those of the lowest quantity are in the GO database. Additionally, 10,122 (65.37%) transcripts were annotated in all five databases (NR, GO, KEGG, Swiss-Prot, and Pfam) (**Figure 3D**).

In the NR database, 14,934 transcripts (96.45%) were annotated. The alignment results of these transcripts with *C. burmanni* revealed that the four species with the highest similarity were *Nelumbo nucifera* (7,157, 20.90%), *Vitis vinifera* (1,272, 8.22%), *Elaeis guineensis* (1,151, 7.43%), and *Phoenix* (817, 5.28%) (**Supplementary Figure 4A**). To uniformly describe gene functions, 10,785 transcripts (69.66%) were subdivided into three GO categories: biological process (BP), cellular component (CC), and molecular function (MF). In the BP category, the top three subgroups were “cellular process” (GO:0009987), “metabolic process” (GO:0008152), and “single-organism process” (GO:0044699). In the CC category, the top three subgroups were “cell” (GO:0005623), “cell part” (GO:0044464), and “organelle” (GO:0043226). In the MF category, the top three subgroups were “binding” (GO:0005488), “catalytic activity” (GO:0003824), and “transporter activity” (GO:0005215) (**Supplementary Figure 4B**).

A total of 14,868 (96.02%) transcripts were successfully annotated in the KEGG database, which were classified into six branches: Cellular Processes, Environmental Information Processing, Genetic Information Processing, Human Diseases, Metabolism, and Organismal Systems, which elucidated the roles and metabolism routes of gene expression products in cells. The five most abundant subgroups were “Signal transduction” (ko02010, ko04016, ko04070, and ko04075) under Environmental Information Processing, “Carbohydrate metabolism” (ko01200) under Metabolism, “Transport and catabolism” (ko04136, ko04144, ko04145, and ko04146) under Cellular Processes, and “Folding, sorting and degradation” (ko03018, ko03050, ko03060, ko04120, ko04122, and ko04141) and “Translation” (ko00970, ko03008, ko03010, ko03013, ko03015, and ko03040) under Genetic Information Processing (**Supplementary Figure 4**C).

**Figure 4:**
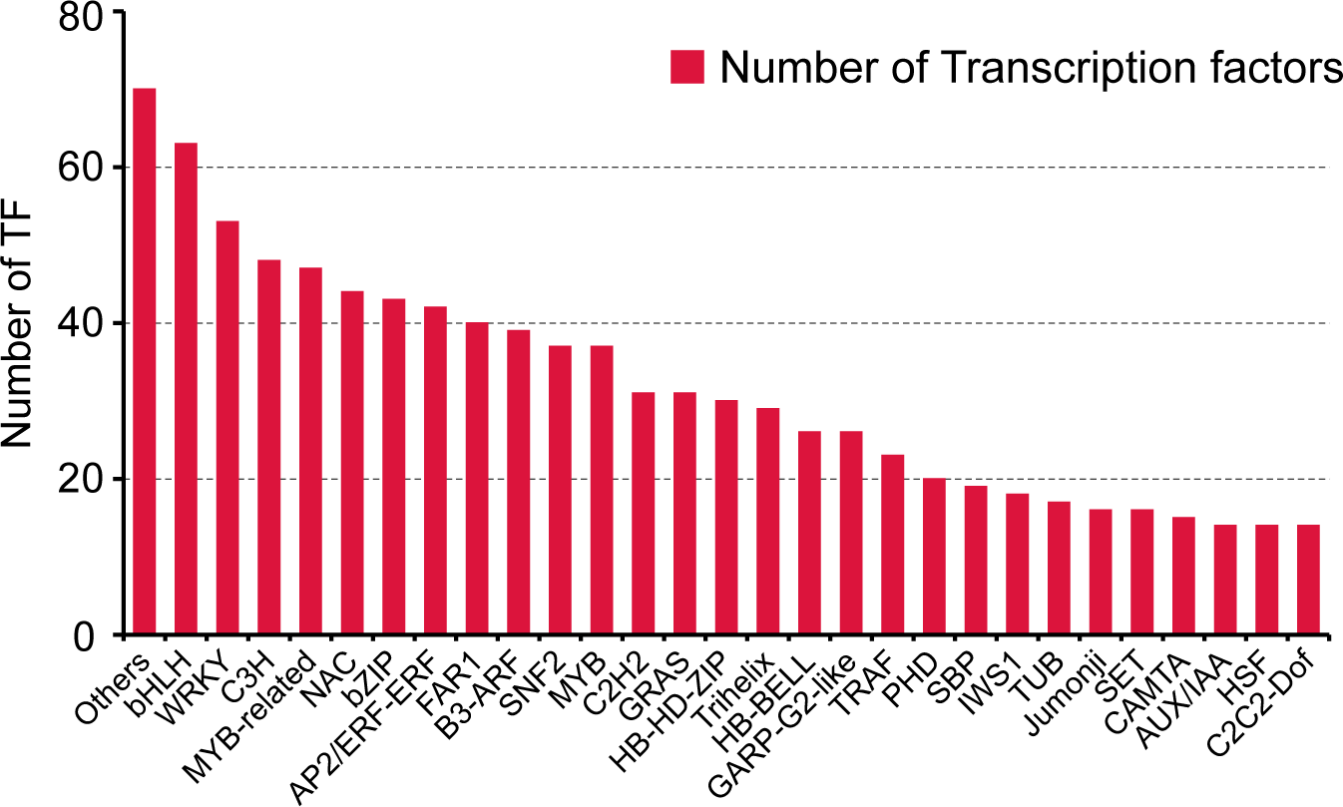
Statistics of transcription factor families identified from full-length transcriptome analysis across differential organs of *Cinnamomum burmanni*.

TFs represent a class of protein molecules that specifically occupy the promoter of target genes to quantitatively modulate the expression of genes with temporal precision and spatial specificity. They currently serve as potent tools widely applied in genetic engineering. We utilized the iTAK software (Zheng et al., 2016) to perform predictive analysis of transcription factors, which identified 922 TFs from 29 gene families (**Figure 4**). The bHLH family exhibited the highest membership (63 members, 6.83%), followed by WRKY (53 members, 5.75%), C3H (48 members, 5.21%), MYB-related (47 members, 5.09%), and NAC families (44 members, 4.77%). In contrast, the AUX/IAA, HSF, and C2C2-Dof families all displayed the lowest representation, which contained 14 members (1.51%). These findings will provide significant implications for the investigation of transcriptional regulation in related biological processes and facilitate the construction of an accurate, high-quality, and comprehensive transcription factor library.

### 3.3 Screening of differentially expressed genes in hormone signal transduction through *C. burmanni* transcriptome analysis

**Figure 5:**
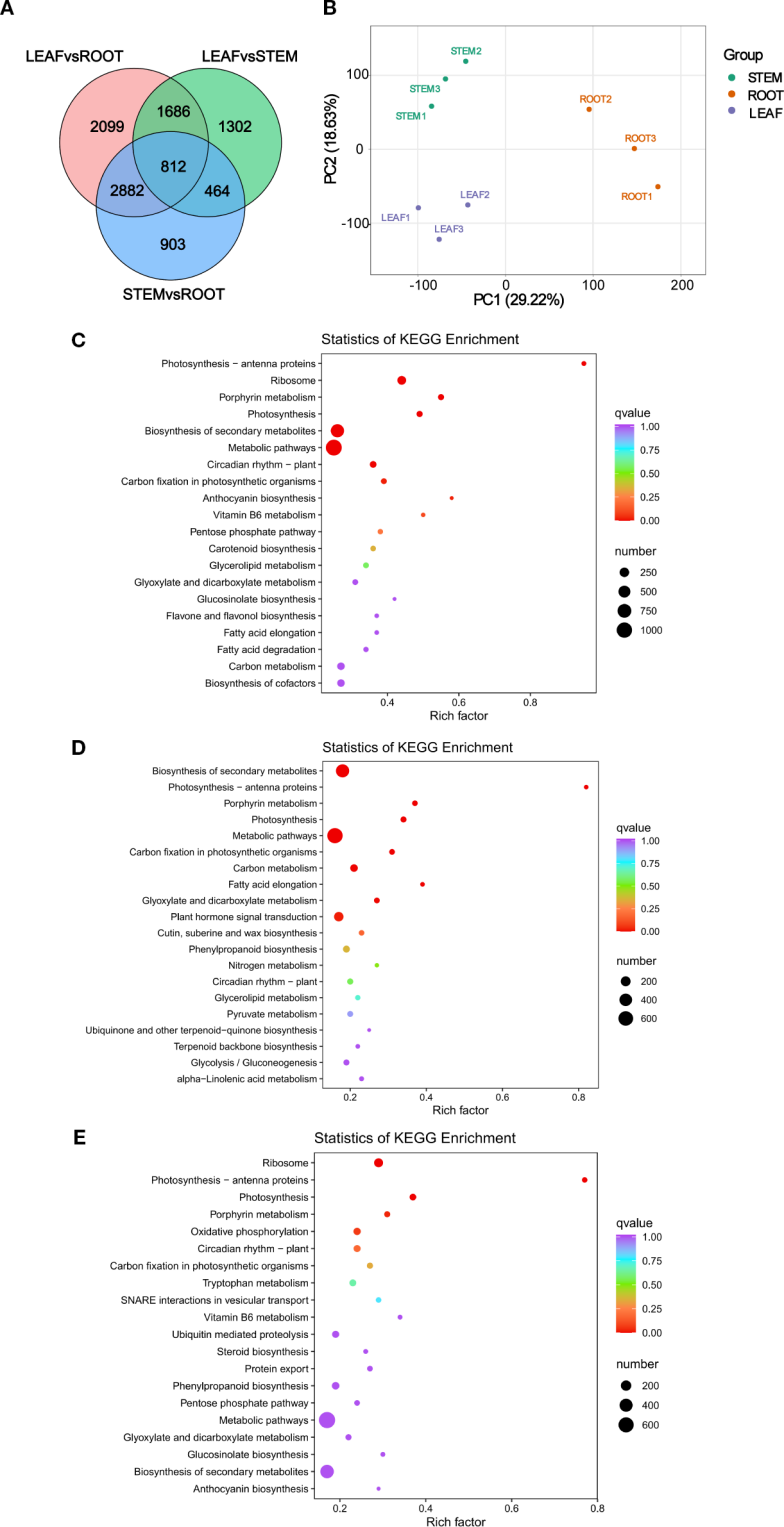
Transcriptomic analysis of *Cinnamomum burmanni*. **(A)** Venn diagram of differentially expressed genes (DEGs) within distinct organs. **(B)** Principal component analysis (PCA) of transcriptomic profiles. **(C)** Kyoto Encyclopedia of Genes and Genomes (KEGG) enrichment statistics of DEGs in ROOTvsLEAF. **(D)** KEGG enrichment plot of DEGs in STEMvsLEAF. **(E)** KEGG enrichment statistics of DEGs in ROOTvsSTEM.

Transcriptome sequencing analysis of RNA samples from three organs was performed to discover the DEGs that are associated with hormone signal transduction in the root, stem, and leaf organs of *C. burmanni*, with three biological repeats, which were performed to confirm the validity of the data. The sequencing yielded raw reads ranging from 40.13 to 47.94 Mb. After quality filtering, error frequency examination, and Guanine-Cytosine (GC) composition allocation assessment, clean reads from the nine cDNA libraries ranged from 38.72 to 47.77 Mb, totaling 61.04 Gb of clean bases, with an average of 6.78 G per sample. The Q20 base percentage for all samples consistently exceeded 97%, and the Q30 base percentage exceeded 93% (**Supplementary Table 6**). For each library, the count of reads that demonstrated successful alignment to the reference genome varied between 30,110,309 (70.76%) and 43,870,063 (93.60%), while uniquely mapped reads ranged from 29,131,403 (68.46%) to 41,971,195 (89.55%) (**Supplementary Table 7**). PCA (**Figure 5B**) demonstrated statistically significant separation among samples from distinct organs, with specimens derived from identical tissues clustering together, which indicated substantial divergence between the three differential groupings. Correlation analysis of *C. burmanni* organs revealed excellent reproducibility across all sample groups (**Supplementary Figure S5A**). Additionally, 812 DEGs were commonly enriched in all three comparative groups (**Figure 5A**). In the ROOTvsLEAF group, 7,479 DEGs exhibited significantly differential expression, including 4,422 upregulated (59.13%) and 3,057 downregulated (40.87%) (**Supplementary Figure S5B**). The STEMvsLEAF group contained 4,264 DEGs, with 1,661 upregulated (38.95%) and 2,603 downregulated (61.05%) (**Supplementary Figure S5C**). The ROOTvsSTEM group displayed 5,061 DEGs, comprising 2,792 upregulated (55.17%) and 2,269 downregulated (44.83%) (**Supplementary Figure S5D**).

The enrichment analyses of GO and KEGG were implemented to elucidate the biological implications of DEGs in this study. The enrichment analysis of GO categorized the functions of DEGs, which demonstrated that 20 GO terms with the greatest enrichment classified into three major GO categories: BP, CC, and MF. Specifically, the BP category contained 14 substantially enriched GO terms, the CC category comprised four, and the MF category included two. Within the three differential groups, the highest quantity of DEGs was enriched in the CC term “cellular anatomical entity” (GO:0110165), followed by the BP term “cellular process” (GO:0009987), the MF term “binding” (GO:0005488), the BP term “metabolic process” (GO:0008152), and the MF term “catalytic activity” (GO:0003824) (**Supplementary Figures 6A–C**). The KEGG pathway analysis provided additional functional insights into the pathways linked to DEGs. The KEGG results revealed that these functional pathways were primarily categorized into five major categories: Cellular Processes, Environmental Information Processing, Genetic Information Processing, Metabolism, and Organismal Systems. Among three distinct groups, the most abundant subpopulations were “Metabolic pathways” (ko01100) and “Biosynthesis of secondary metabolites” (ko01110) under the Metabolism category, as well as “Plant−pathogen interaction” (ko04626) under the Organismal Systems category (**Supplementary Figures 7A–C**). KEGG enrichment analysis revealed that the DEGs were predominantly enriched in the following pathways: “Biosynthesis of secondary metabolites” (ko01110), “Photosynthesis” (ko00195), “Photosynthesis-antenna proteins” (ko00196), and “Porphyrin metabolism” (ko00860) (**Figures 5C–E**).

### 3.4 Identification of differentially expressed genes in plant hormone signal transduction via WGCNA

**Figure 6:**
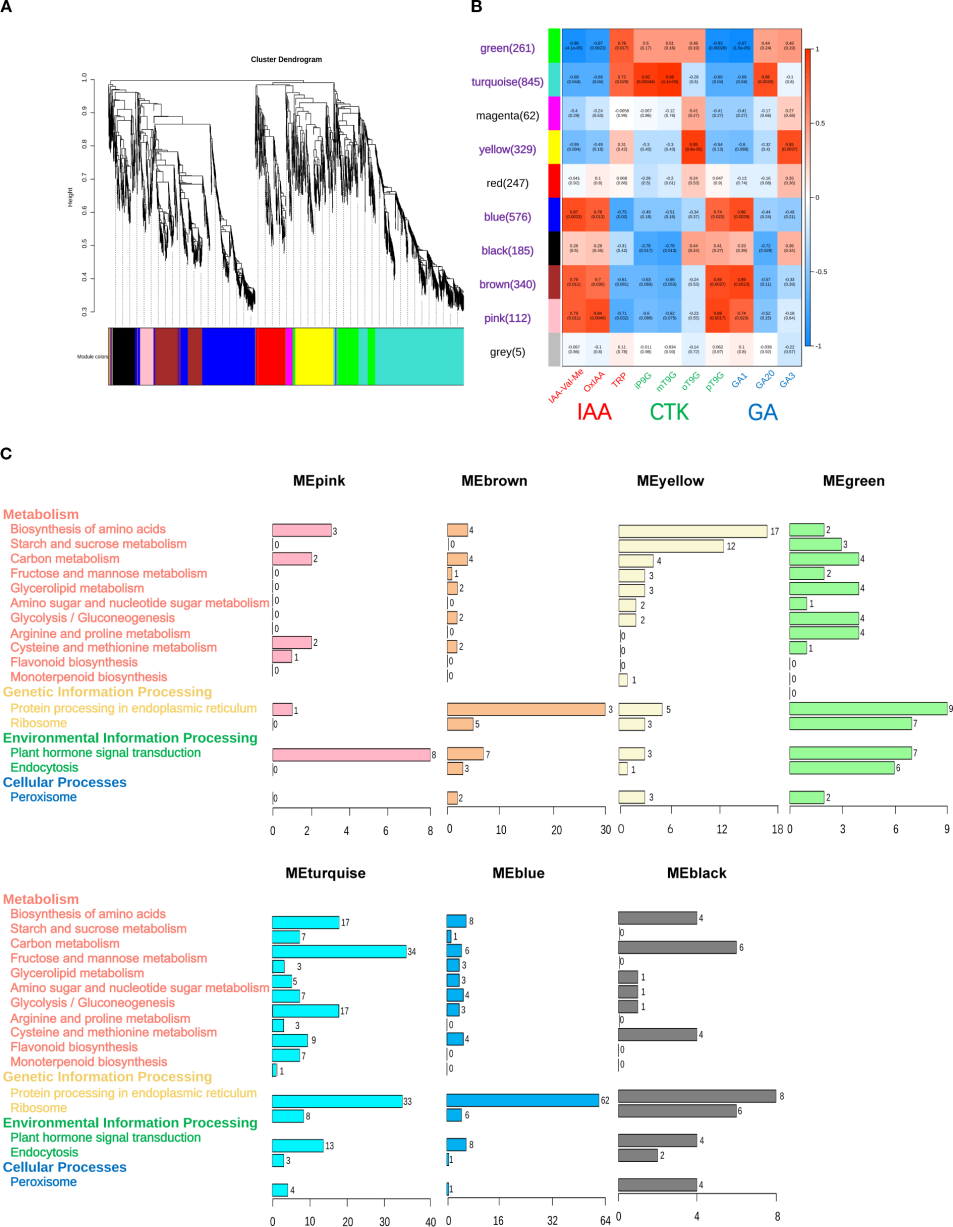
Weighted gene co-expression network analysis (WGCNA) results of differentially expressed genes (DEGs) associated with 10 specific hormones in *Cinnamomum burmanni*. **(A)** Hierarchical clustering dendrogram of gene modules. **(B)** Heatmap of correlations between DEG modules and traits. The red labels represent indole-3-acetic acid (IAA) hormones on x-axis, green labels represent cytokinin (CTK) hormones, blue labels represent gibberellin (GA) hormones, and purple labels represent seven high correlation modules. **(C)** Kyoto Encyclopedia of Genes and Genomes (KEGG) pathway analysis of significantly correlated modules in highly correlated modules.

To investigate the DEGs that are associated with hormone signal transduction across differential organs, this study selected 10 highly expressed hormones from multiple organs, including three IAA hormones (IAA-Val-Me, OxIAA, and TRP), three GA hormones (GA1, GA3, and GA20), and four CTK hormones (iP9G, mT9G, oT9G, and pT9G). Based on transcriptome profiling data, WGCNA was applied with a filtering threshold set at 0.85 (85% of genes were excluded, retaining 15% of the data for analysis). By applying hierarchical clustering analysis, all genes were partitioned into 10 modules, each containing genes with similar expression patterns and labeled with distinct colors (**Figure 6A**). The five largest modules were turquoise (845 genes), blue (576 genes), brown (340 genes), yellow (329 genes), and green (261 genes) (**Figure 6B**).

Seven modules exhibiting significant correlations with the hormone signaling pathway were identified from the WGCNA results. Specifically, the green module demonstrated a significantly positive correlation with TRP (p < 0.01) and displayed strongly negative correlations with GA1, IAA-Val-Me, OxIAA, and pT9G (p < 0.01). The turquoise module demonstrated a positive correlation with TRP (p < 0.05); exhibited significantly positive correlations with GA20, iP9G, and mT9G (p < 0.01); and displayed negative correlations with GA1, OxIAA, and pT9G (p < 0.05) and a strongly negative correlation with IAA-Val-Me (p < 0.01). The yellow module demonstrated a significantly positive correlation with GA3 and oT9G (p < 0.01). The blue module displayed positive correlations with OxIAA and pT9G (p < 0.05), exhibited strongly positive correlations with GA1 and IAA-Val-Me (p < 0.01), and demonstrated a negative correlation with TRP (p < 0.05). The black module exhibited negative correlations with GA20, iP9G, and mT9G (p < 0.05). The brown module displayed significantly positive correlations with GA1 and pT9G (p < 0.01) and exhibited positive correlations with IAA-Val-Me and OxIAA (p < 0.05). The pink module demonstrated strongly positive correlations with OxIAA and pT9G (p < 0.01), exhibited positive correlations with GA1 and IAA-Val-Me (p < 0.05), and displayed a negative correlation with TRP (p < 0.05) (**Figure 6B**). Furthermore, seven significantly correlated modules were subjected to the KEGG pathway analysis using the graphics package of R (version 3.5.1). The results demonstrated that the turquoise module (13 genes) contained the highest count of DEGs connected to hormone signal transduction, whereas the yellow module (three genes) exhibited the lowest quantity of DEGs associated with hormone signaling pathways (**Figure 6C**).

### 3.5 Integrated analysis of differentially expressed genes of hormone signaling pathway and differential hormones

**Figure 7:**
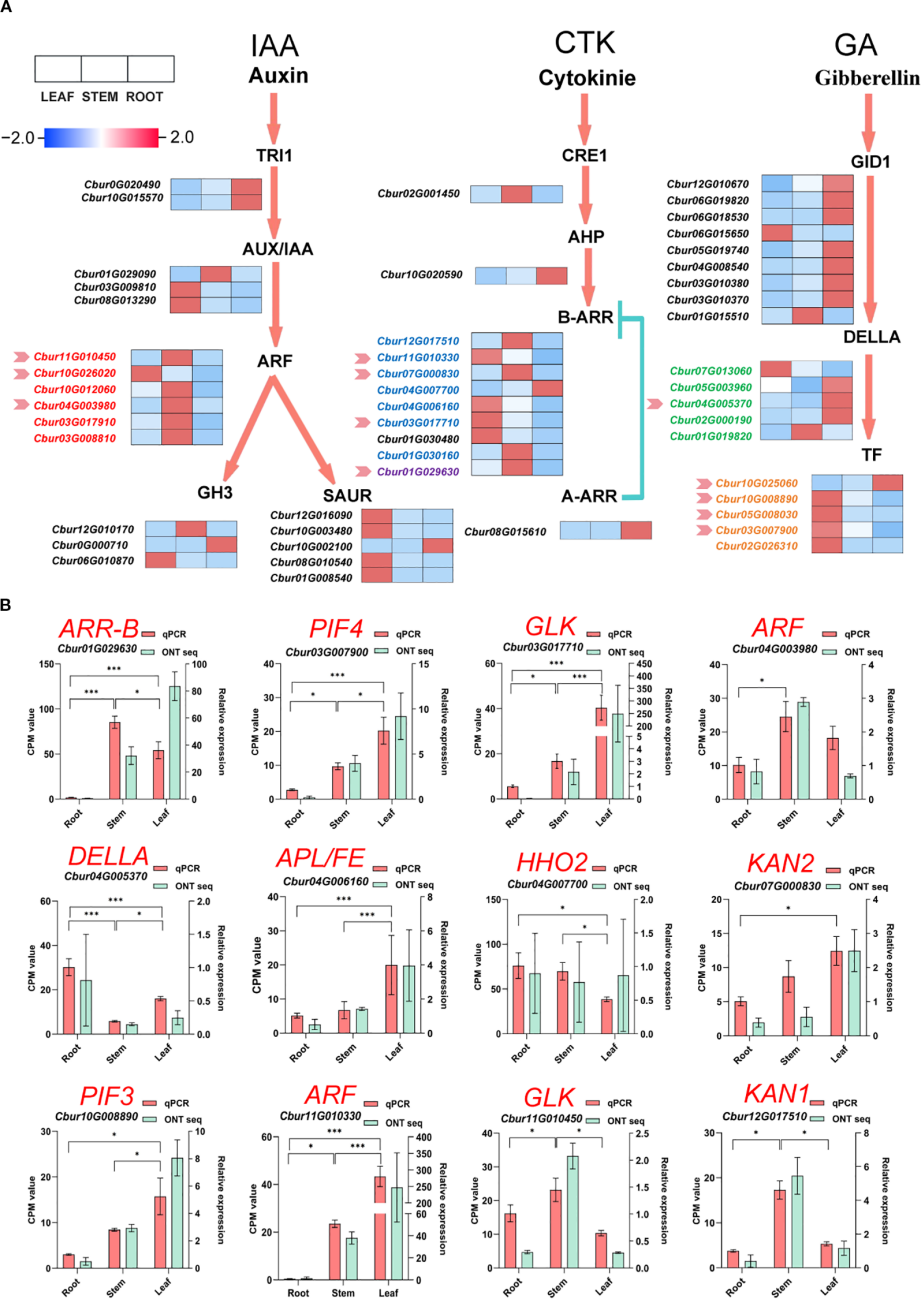
**(A)** Heatmap of differentially expressed genes (DEGs) in plant hormone signal transduction and metabolic regulation pathways. Red demonstrates high expression level in the organ, while blue represents low expression level. DEG names in red denote association with ARF family transcription factors, orange with bHLH family, green with GRAS family, blue with G2-like family, and purple with ARR-B family. Pink arrows indicate genes validated by qRT-PCR experiments. **(B)** Quantitative real-time polymerase chain reaction (qRT-PCR) used ACTIN as the internal reference control. Symbols * and *** indicate p-value < 0.05 and p-value < 0.01, respectively, as determined by one-way ANOVA followed by t-test.

Transcriptomic and metabolomic analyses were integrated to construct a heatmap, which was linked to phytohormone signal transduction and metabolic regulation pathways to explore DEGs across three organs of *C. burmanni*. A total of 50 DEGs related to hormone signal transduction were determined (**Supplementary Table 8**). Specifically, 19 DEGs were linked to auxin, with eight upregulated in leaves, seven in stems, and four in roots; 12 DEGs were associated with cytokinin, including four with highly expressed levels in leaves, five in stems, and three in roots; 19 DEGs were related to gibberellin, among which six exhibited elevated expression in leaves, two in stems, and 11 in roots (**Figure 7A**). Twelve candidate DEGs were randomly chosen to perform qRT-PCR assays for validating the patterns of gene expression. The experimental results confirmed consistency between the observed expression profiles and bioinformatics predictions (**Figure 7B**).

### 3.6 Correlation analysis of DEGs among *C. burmanni*’s differential organs and their relationship with phytohormones

**Figure 8:**
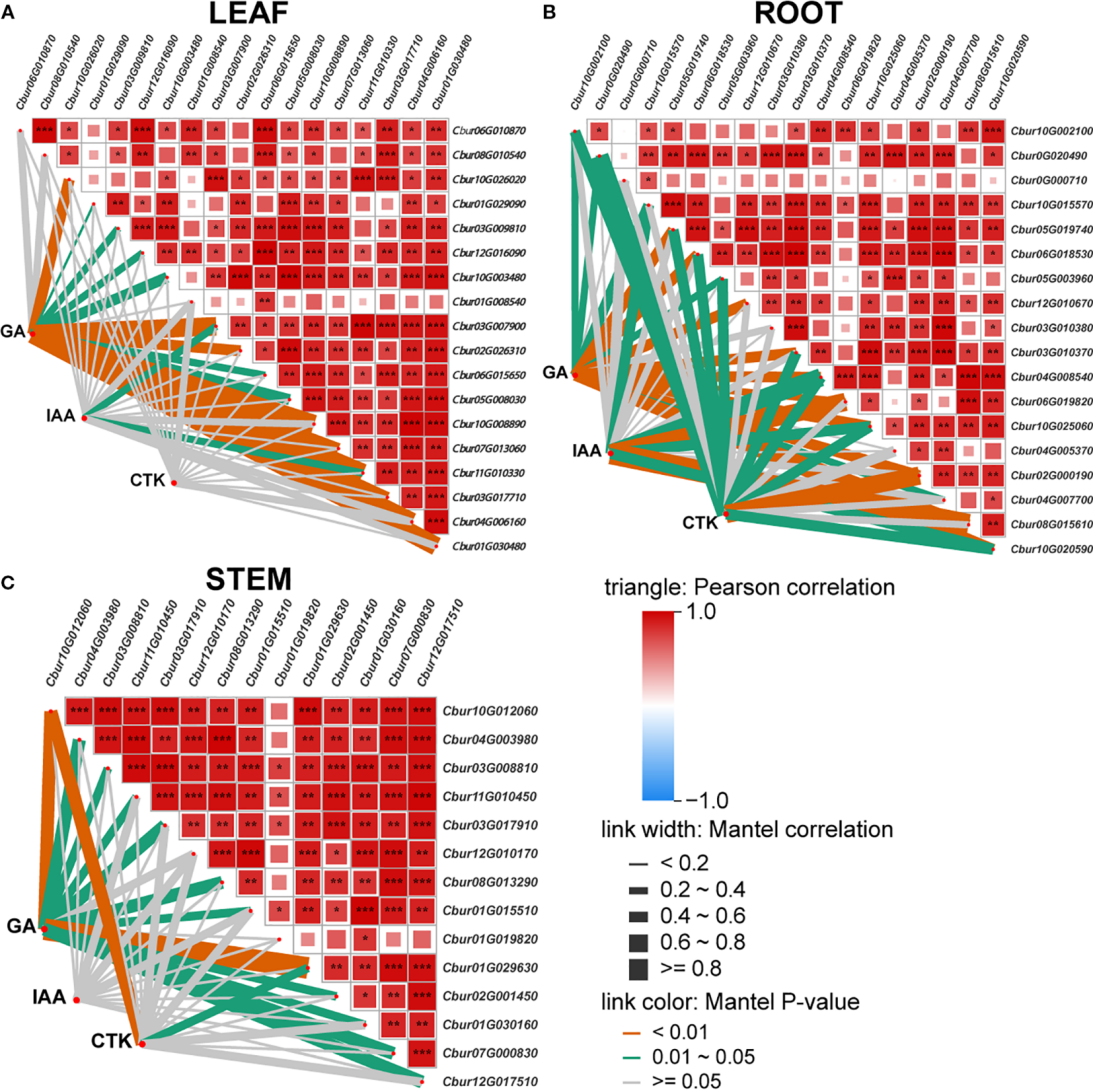
The Mantel test correlation heatmap. The pairwise comparisons of hormone signal transduction differentially expressed genes; the color gradient indicates Spearman’s correlation coefficients. Partial (geographic distance-corrected) Mantel test showing correlations among indole-3-acetic acid (IAA), gibberellin (GA), cytokinin (CTK), and organ [leaf **(A)**, root **(B)**, and stem **(C)**] differentially expressed genes. Line thickness indicates correlation strength; color represents significance (red, positive; blue, negative).

The Mantel test correlation heatmap analysis revealed that the association patterns of three phytohormones (IAA, CTK, and GA) with significantly DEGs in the root, stem, and leaf organs exhibited remarkable tissue specificity. In leaves (LEAF), GA demonstrated the most prominent correlation effects, showing highly significant associations with nine DEGs (p < 0.01) and significant correlations with eight DEGs (p < 0.05), with the number of significantly correlated genes substantially exceeding that of IAA and CTK. IAA exhibited significant correlations with only two genes (p < 0.05) (**Figure 8A**). In roots (ROOT), both GA and CTK displayed extensively regulatory capacities. GA correlated with 10 DEGs at highly significant levels (p < 0.01) and five DEGs at significant levels (p < 0.05), while CTK showed one DEG at highly significant levels (p < 0.01) and nine DEGs with significant correlations (p < 0.05). The number of IAA-associated genes was fewer, with only six exhibiting significant/highly significant levels (**Figure 8B**). In stems (STEM), GA dominated the regulatory network, which exhibited highly significant correlations with 10 DEGs (p < 0.01) and significant associations with two DEGs (p < 0.05). CTK displayed significant correlations with only two genes (p < 0.05), but IAA showed no significant DEG associations (**Figure 8C**).

### 3.7 Co-expression network comprehensive analysis of differentially expressed genes, transcription factors, and phytohormones

The DEGs that were associated with the three hormones (IAA, CTK, and GA) were employed to predict and screen TFs using PlantTFDB v5.0 (Tian et al., 2020) (**Supplementary Table 9**). Among these, TRI1, AUX/IAA, GH3, SAUR, CRE1, AHP, A-ARR, and GID1 were excluded during the prediction process since they function as auxiliary regulatory proteins instead of transcription factors. The prediction results encompassed five transcription factor families, including ARF, bHLH (PIF3/4), G2-like (DELLA), GRAS (GLK/KAN1/2/HH2O/APL/FT), and ARR-B.

**Figure 9:**
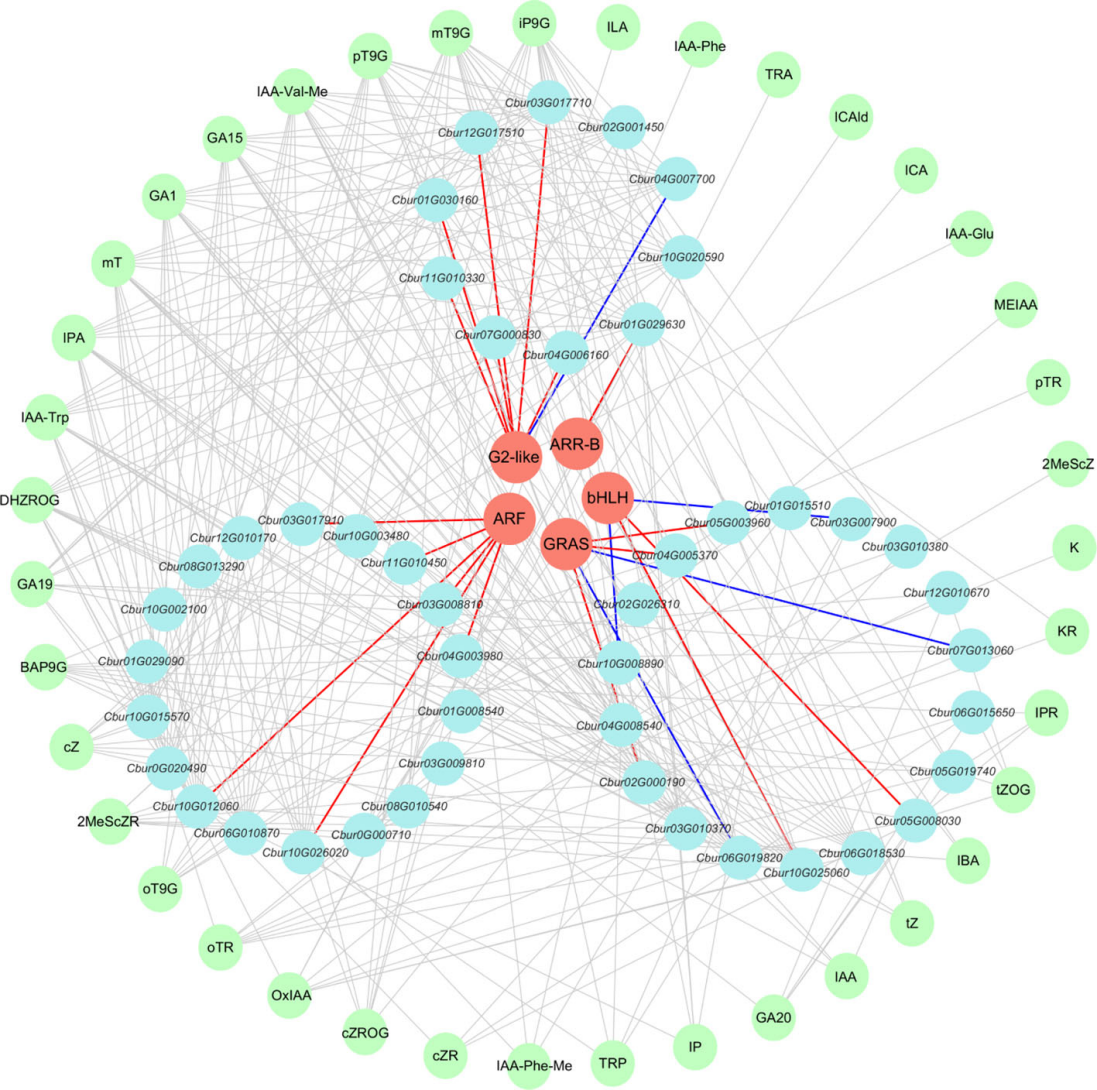
Co-expression network analysis of differentially expressed genes (DEGs), transcription factors (TFs), and phytohormones. Salmon circles denote predicted TFs; light blue circles denote identified phytohormones; light yellow circles denote identified DEGs. Red edges represent positive correlations between DEGs and TFs; blue edges represent negative correlations between DEGs and TFs.

To investigate the relationships among DEGs, phytohormones, and TFs in the IAA, GA, and CTK signaling pathways, correlation analysis was conducted. The screening criteria for analysis were established at p < 0.05, and the absolute correlation coefficient surpassed 0.8. The results revealed 39 differential phytohormones and 46 DEGs. Specifically, 15 phytohormones and 18 DEGs were associated with IAA, four phytohormones and 18 DEGs were linked to GA, and 20 phytohormones and 10 DEGs were correlated with CTK. Among the six DEGs participating in the IAA signaling pathway, the expression of *Cbur03G008810*, *Cbur03G017910*, *Cbur04G003980*, *Cbur10G012060*, *Cbur10G026020*, and *Cbur11G010450* exhibited positive correlations with the ARF transcription factor. For the nine DEGs engaged in the GA signaling pathway, *Cbur05G008030* and *Cbur10G025060* showed positive correlations with the bHLH transcription factor, whereas *Cbur03G007900* and *Cbur10G008890* displayed negative correlations with bHLH. Additionally, *Cbur02G000190*, *Cbur04G005370*, and *Cbur05G003960* demonstrated positive correlations with the GRAS transcription factor, while *Cbur01G019820* and *Cbur07G013060* exhibited negative correlations with GRAS. Among the eight DEGs that participated in the CTK signaling pathway, *Cbur12G017510*, *Cbur11G010330*, *Cbur01G030160*, *Cbur03G017710*, *Cbur07G000830*, and *Cbur04G006160* showed positive correlations with G2-like, whereas *Cbur04G007700* displayed a negative correlation with the ARR-B transcription factor. Conversely, *Cbur01G029630* exhibited a positive correlation with ARR-B (**Figure 9**).

## 4 Discussion

Hormones play a key role in growth and secondary metabolism. Through integrated metabolomic and transcriptomic analyses of *C. burmanni*, this study reveals organ-specific hormonal signatures and signaling pathways for root, stem, and leaf development. The main findings show that auxin (IAA) mainly accumulates in leaves, CTK accumulates in stems, and GA has a dynamic tissue-specific distribution, which is related to the differential expression of hormone signaling genes (e.g., ARF, Aux/IAA, DELLA, and ARR-B) in different organs. Importantly, the research identifies a complex transcription factor network (including ARF, DELLA, PIF3/4, and ARR-B families) that can modulate organ development and potentially coordinate terpenoid biosynthesis (e.g., borneol) via hormone signaling crosstalk. These results establish a molecular framework connecting hormone dynamics with morphology and specialized metabolism, providing basic knowledge for improving stress resilience and bioactive compound production in this economically important species.

Auxin, as a master regulator of plant growth, participates in regulating cell elongation promotion, apical dominance maintenance, and root differentiation (Woodward and Bartel, 2005). Our study demonstrates that auxin hormones are highly concentrated in leaves (**Figure 2D**, **Supplementary Table 4**). Observing auxin signaling genes exhibiting organ-specific expression profiles in *C. burmanni* (**Figure 7A**), we speculated that this phenomenon may be associated with the DEGs (*ARF*, *Aux/IAA*, *GH3*, and *SAUR*) in leaves (**Figure 7A**). As previously reported, auxin signal transduction regulates the development of plants through ARF and Aux/IAA, modulating the expression of downstream genes (Li et al., 2023a). Mei et al. (2022) described that *MsARF5* mediates the auxin signaling pathway to increase *Magnolia sieboldii* plant height, stem diameter, and leaf width. Xu et al. (2022) reported that the elevated expression of *CgARF1* accelerated leaf aging and diminished the content of chlorophyll in *Cymbidium goeringii*. Li et al. (2023a) revealed that ARF1 modulates the transcriptional process of YUC2-mediated auxin biosynthesis through the auxin signaling pathway. Seventeen auxin (IAA) signal transduction genes involved in the regulation of adventitious root formation in *C. bodinieri* have been identified (Yu et al., 2025). Luo et al. (2025) indicated that IAA biosynthesis and its signaling pathways control the development of adventitious buds and roots of *C. parthenoxylon*. Therefore, we hypothesized that the coordinated high expression of *ARF*s and their *Aux/IAA* repressors may function as critical regulators, affecting leaf development, including the expression of hormone biosynthesis genes in the leaves to trigger high concentrations of hormones.

GA participates in regulating multiple developmental processes in plants, such as photomorphogenesis (Alabadí et al., 2004) and stress responses (Navarro et al., 2008). Our research indicates that GA hormonal disparities across different organs are not substantial (**Figure 2D**, **Supplementary Table 4**). We speculate that it may be associated with the *GDI1* and *DELLA* genes exhibiting highly similar organ-specific expression patterns in *C. burmanni* (**Figure 7A**), as well as the GA-GID1-DELLA module in GA signal transduction. GA signal transduction modulates plant development by DELLA transcription factors and other proteins that regulate downstream gene expression (Phokas and Coates, 2021; Xue et al., 2022). The receptor GID1 perceives GA, which subsequently interacts with the DELLA protein and facilitates its degradation to alter transcription factor activity (Hernández-García et al., 2021). Guan et al. (2021) reported that DELLA proteins negatively regulate the GA signaling pathway to influence stem elongation and bolting processes in *Brassica campestris*. Ritonga et al. (2023) revealed that DELLA negatively regulates cell elongation, leaf senescence, and leaf head formation by suppressing GA biosynthesis gene expression. Thus, we speculated that strong expression of *GID1*s and *DELLA*s in some organs may regulate the growth and development of these organs by modulating the expression of GA biosynthesis genes to influence the GA signal transduction pathway and GA content. Furthermore, based on our research, the DEGs of “TF” in the GA signaling pathway were predicted as belonging to the PIF family (*PIF3*/*PIF4*) (**Supplementary Table 9**). Root tissues and leaves exhibited DELLA-PIF-mediated signaling (**Figure 7A**). Li et al. (2016) revealed that DELLA proteins in *A. thaliana* inhibit PIF3 and PIF4 by promoting ubiquitin-proteasome degradation. Guo et al. (2024b) reported that PIF3 or PIF4 can interplay with DELLA proteins that suppress GA signal transduction to modulate hypocotyl growth. Therefore, we hypothesized that these genes may participate in the developmental regulation of roots and leaves via interaction, leading to the higher expression.

Cytokinins are involved not only in plant growth (Kieber and Schaller, 2018) but also in responses to biotic and abiotic stresses (Cortleven et al., 2019). This investigation demonstrates that CTK hormones are highly concentrated in stems (**Figure 2D**, **Supplementary Table 4**), exhibiting CTK signaling genes that possessed organ-specific transcriptional profiles in *C. burmanni* (**Figure 7A**). We speculated that the elevated levels of cytokinin hormones in stems may be associated with the DEGs (*B-ARR*) in the stems (**Figure 7A**). The regulation of plant development by the CTK signaling pathway may be associated with the ARR-B transcription factors and ARR-A modulator proteins (Lai et al., 2021). ARR-As, which are activated by ARR-Bs, compete with ARR-Bs for phosphorylation signal to negatively modulate the CTK signaling pathway (Hwang and Sheen, 2001; Tan et al., 2019). In *A. thaliana*, *ARR10*, *ARR12*, and *ARR18* of type B cause an increased quantity of mutant plants of bud regeneration, hypocotyl elongation, and development of axillary meristem (Zhu et al., 2022). Ou et al. (2022) revealed that ARR1-b positively regulates cytokinin signal transduction to promote root and stem elongation and inhibit leaf senescence and stem thickening in Flowering Chinese cabbage (*Brassica rapa*). Thus, we supposed that high expression of *ARR-B*s in stems may regulate the growth and development of stems by transmitting the signal of CTK to influence the CTK hormone level.

As an essential oil-producing plant, *C. burmanni*’s terpenoid biosynthesis is potentially regulated by hormone signaling pathways. Previous studies have shown that ARF-related genes (including miRNAs and ARF transcription factors) of auxin signaling and DELLA of GA signaling participate in terpenoid metabolism. Wang et al. (2025b) reported that *HcARF8* and the auxin receptor *HcTIR1* negatively regulate floral scent compound synthesis in *Hedychium coronarium*, with *HcARF8* directly binding to the promoter of the terpene synthase gene *HcTPS8* to modulate terpenoid biosynthesis. Zhang et al. (2020) demonstrated that miR167 targets the ARF6 transcription factor to regulate *TPS1* and *TPS5* expression in *H. coronarium*, influencing terpenoid biosynthesis. Hong et al. (2012) demonstrated that GA signaling repressor DELLA inhibits the function of sesquiterpene synthase genes in *A. thaliana* (including TPS11 and TPS21) via interaction with MYC2 transcription factor. Notably, Danova et al. (2018) observed that CTK treatment boosts sesquiterpene production in *Artemisia alba* Turra. Therefore, with the treatment of CTK, the identified *ARF* and *DELLA* gene families may be involved in modulating terpenoid biosynthesis in *C. burmanni*.

## 5 Conclusions

**Figure 10:**
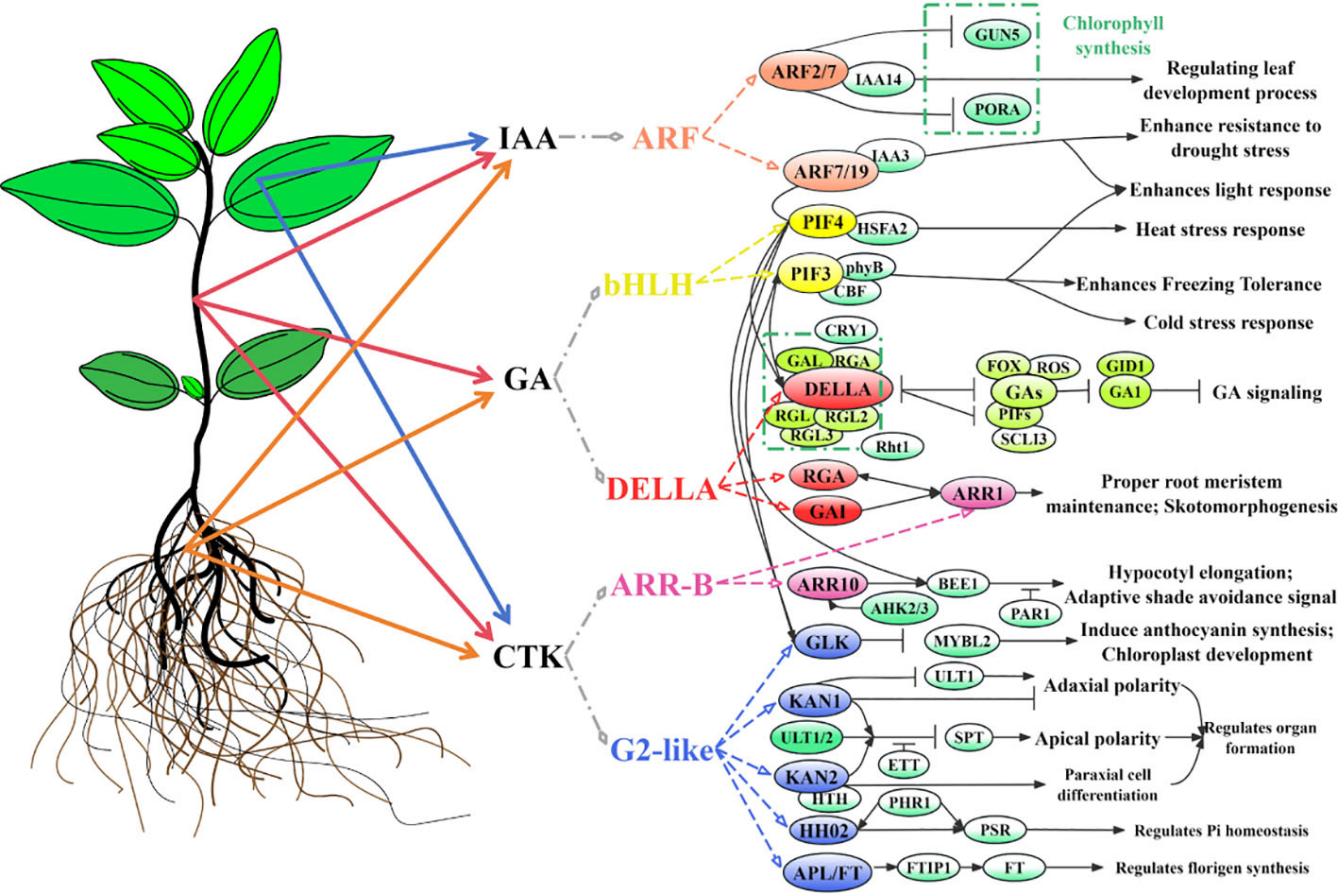
Regulatory model diagram of hormones and predicted transcription factors (TFs) in differential organs of *Cinnamomum burmanni*. Chartreuse represents TFs and proteins in *Solanum lycopersicum*, spring green represents TFs and proteins in *Arabidopsis thaliana*, and other colors represent predicted TFs in *C. burmanni*.

This study integrated metabolomics, transcriptomics, and full-length transcriptomics to elucidate the molecular mechanisms underlying the hormone distribution and expression regulation of genes in differential organs (roots, stems, and leaves) of *C. burmanni*. Metabolomic data revealed 70 differential hormones across the three organs, with IAA hormones significantly enriched in leaves, while GA and CTK hormones were highly expressed in stems. This distribution pattern was closely linked to the regulatory modes of organ-specific DEGs discovered by the transcriptomic data (**Supplementary Table 8**). Utilizing transcriptomics and full-length transcriptomics, we identified 812 key DEGs, including 50 related to hormone signal transduction, and we validated 12 DEGs through qPCR experiments. This study identified 10 transcription factors that participate in hormone signal transduction, highlighting the central roles of ARF, bHLH (PIF3/4), GRAS (DELLA), G2-like (GLK/KAN1/2/HH2O/APL/FT), and ARR-B transcription factors in the hormone signaling pathway. These factors belonged to five gene families, forming a molecular regulatory network of hormones, genes, and TFs (**Figures 10**). The discovery of these DEGs and TFs further clarified the regulatory role of hormone signaling networks in organ development and environmental adaptation. Future research could employ gene editing or transgenic technologies to verify the roles of these pivotal genes and investigate the latent applications of hormone interaction networks in stress resistance breeding of *C. burmanni* using multi-omics data.

## Data Availability

The data of full-length sequencing presented in the study are deposited in the NCBI Sequence Read Archive (SRA), accession number PRJNA1287254. The transcriptomic data presented in the study are deposited in the NCBI Sequence Read Archive (SRA), accession number PRJNA1286930.
