# Supplementary material for: Multi-omics analysis of organ-specific hormone distribution and molecular regulatory mechanisms in Cinnamomum burmanni
Source: Front Plant Sci. 2025 Sep 19;16:1662457. doi: 10.3389/fpls.2025.1662457 (PMC12491295; doi:10.3389/fpls.2025.1662457)
Supplement: Supplementary file 1 [file DataSheet1.zip › Supplementary Figure 2.pdf]

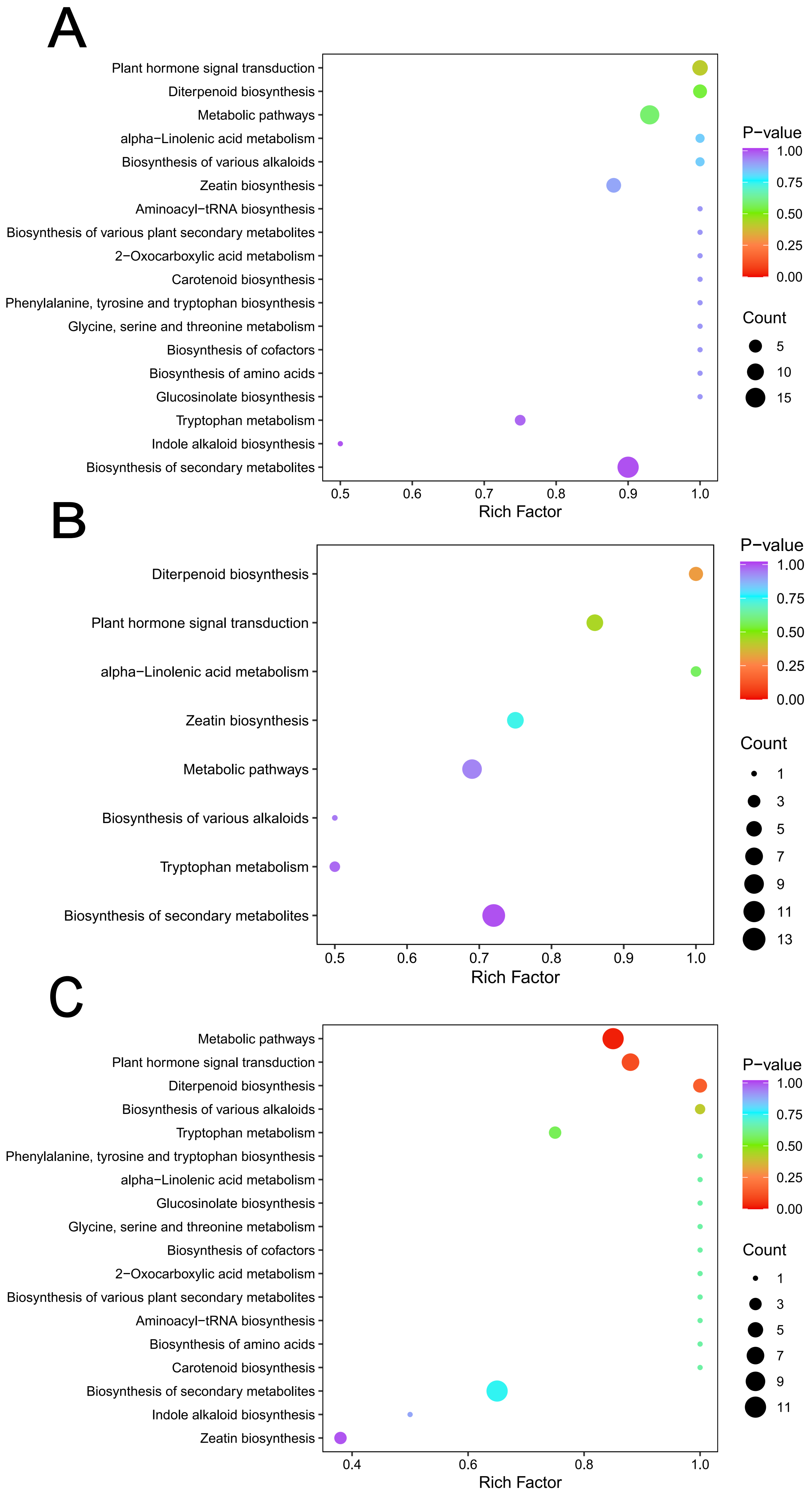

Supplementary Figure 2. KEGG analysis of the significantly differential hormones in three organs. (A) KEGG enrichment statistics of significantly differential hormones in ROOTvsLEAF. (B) KEGG enrichment plot of significantly differential hormones in STEMvsLEAF. (C) KEGG enrichment statistics of significantly differential hormones in ROOTvsSTEM.
