# Supplementary material for: Multi-omics analysis of organ-specific hormone distribution and molecular regulatory mechanisms in Cinnamomum burmanni
Source: Front Plant Sci. 2025 Sep 19;16:1662457. doi: 10.3389/fpls.2025.1662457 (PMC12491295; doi:10.3389/fpls.2025.1662457)
Supplement: Supplementary file 1 [file DataSheet1.zip › Supplementary Figure 3.pdf]

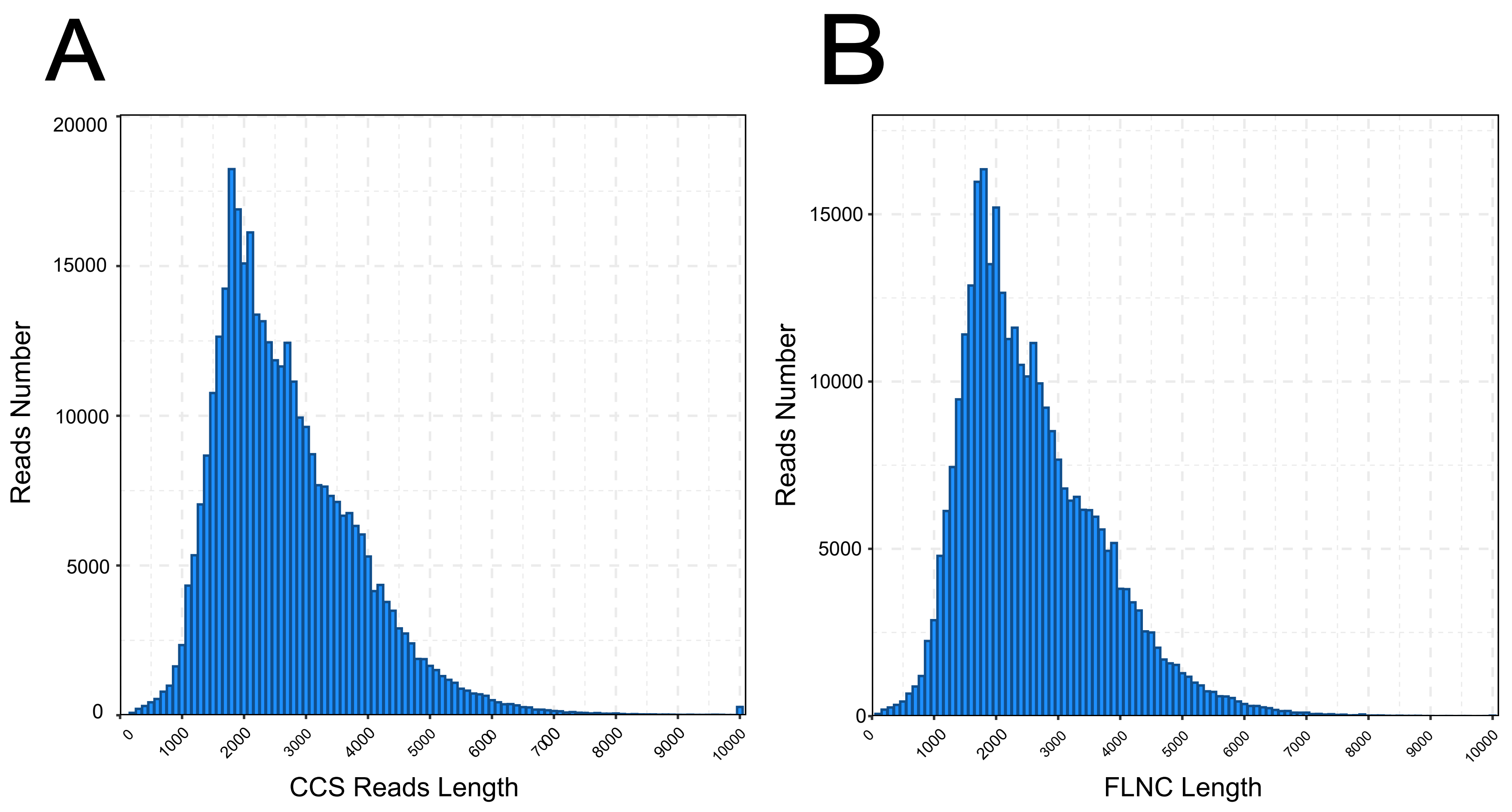

Supplementary Figure 3. (A)The length distribution of CCS sequences. (B)The distribution of FLNC sequence lengths.
