# Supplementary material for: Multi-omics analysis of organ-specific hormone distribution and molecular regulatory mechanisms in Cinnamomum burmanni
Source: Front Plant Sci. 2025 Sep 19;16:1662457. doi: 10.3389/fpls.2025.1662457 (PMC12491295; doi:10.3389/fpls.2025.1662457)
Supplement: Supplementary file 1 [file DataSheet1.zip › Supplementary Figure 4.pdf]

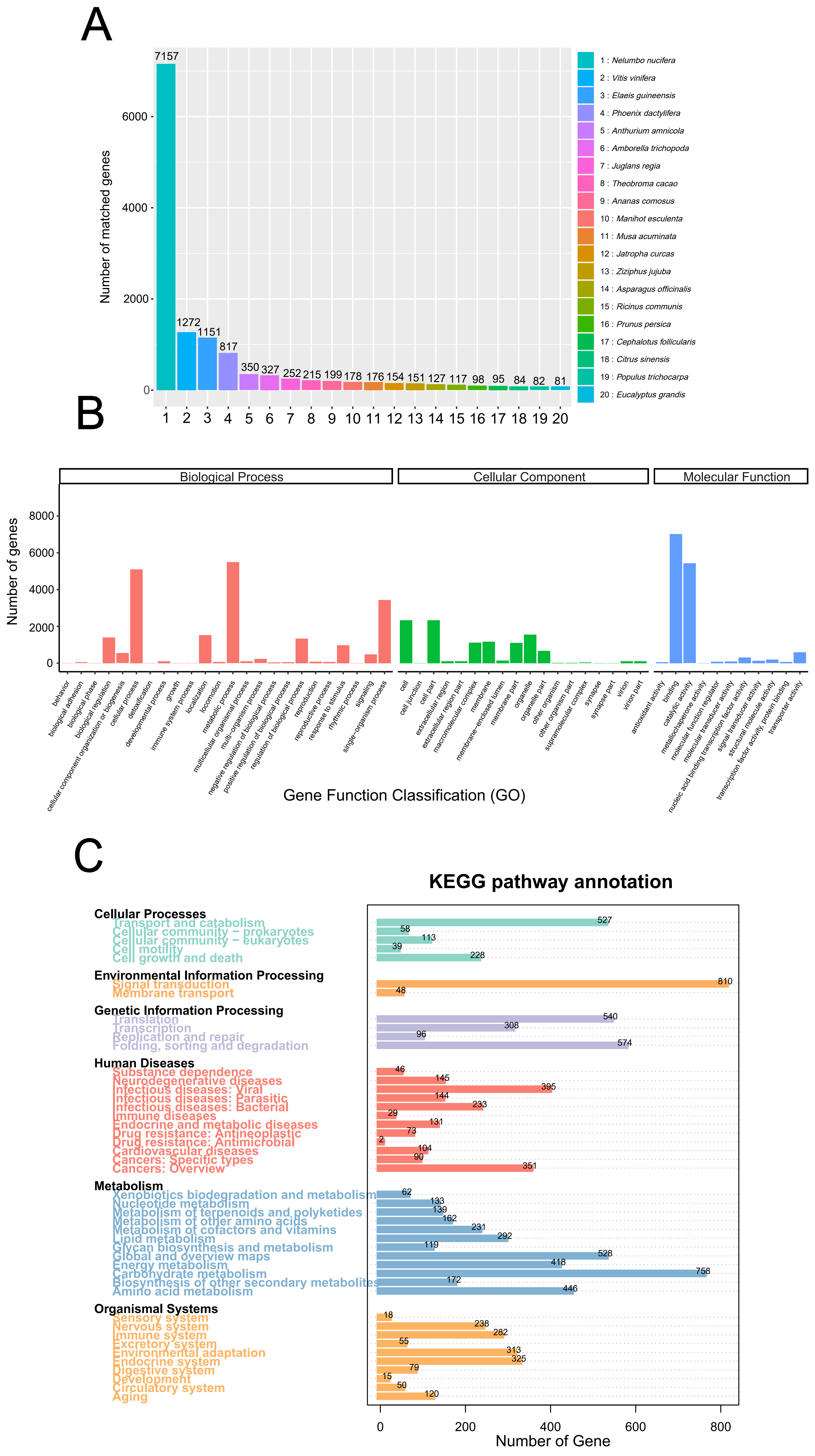

Supplementary Figure 4. Functional annotation of structurally annotated transcripts in Non-Redundant Protein Database (NR), Gene Ontology (GO) and Kyoto Encyclopedia of Genes and Genomes (KEGG) transcripts (A)NR classification of the assembled full-length transcripts. (B)GO classification of the assembled full-length transcripts. (C)KEGG annotation of the assembled full-length transcripts.
