# Supplementary material for: Multi-omics analysis of organ-specific hormone distribution and molecular regulatory mechanisms in Cinnamomum burmanni
Source: Front Plant Sci. 2025 Sep 19;16:1662457. doi: 10.3389/fpls.2025.1662457 (PMC12491295; doi:10.3389/fpls.2025.1662457)
Supplement: Supplementary file 1 [file DataSheet1.zip › Supplementary Figure 6.pdf]

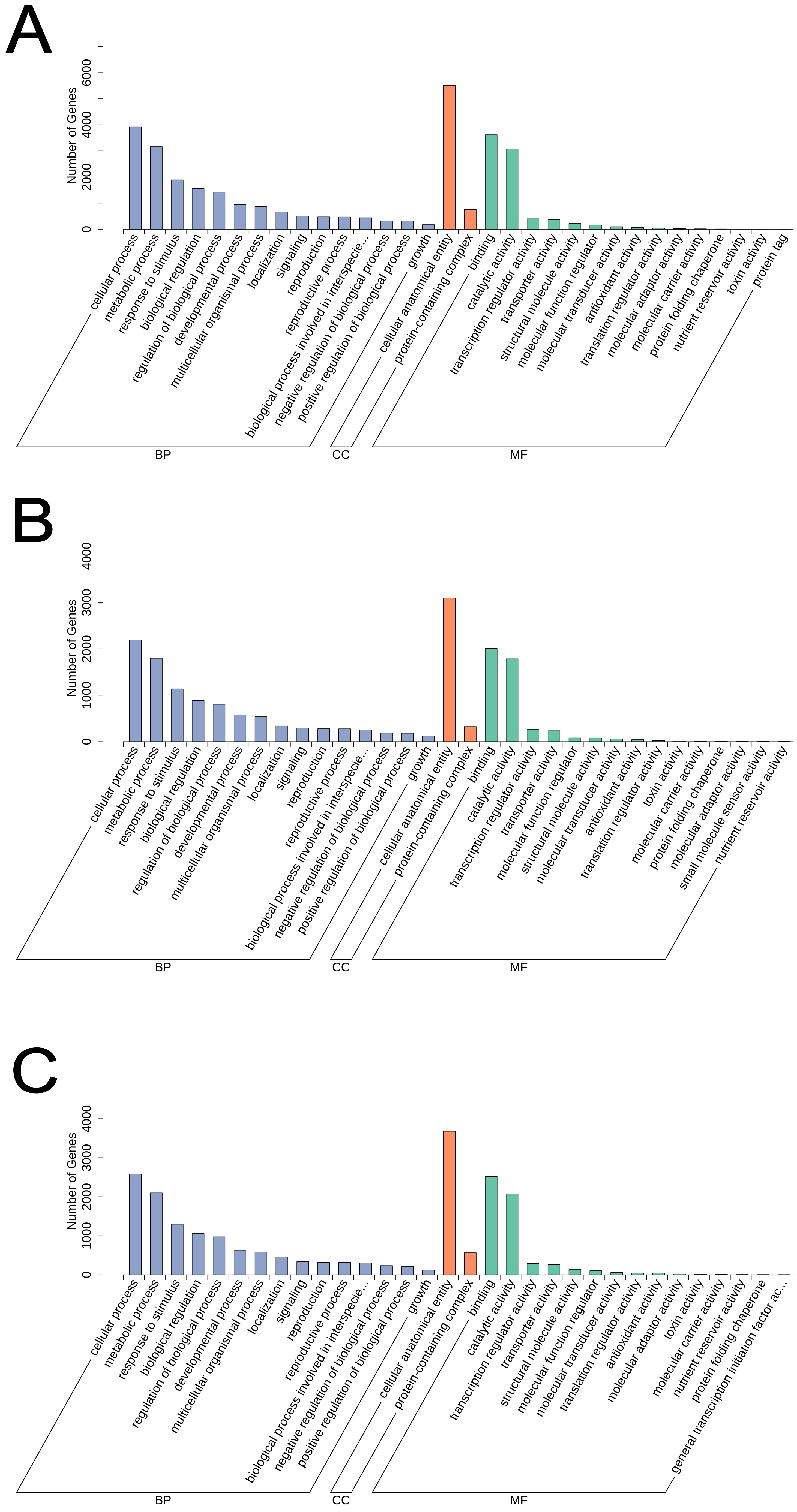

Supplementary Figure 6. Gene Ontology (GO) enrichment analysis of differentially expressed genes (DEGs) in *Cinnamomum burmannii*. (A) GO enrichment of ROOT vs LEAF group, (B) GO enrichment of STEM vs LEAF group, (C) GO enrichment of ROOT vs STEM group.
