# Supplementary material for: Multi-omics analysis of organ-specific hormone distribution and molecular regulatory mechanisms in Cinnamomum burmanni
Source: Front Plant Sci. 2025 Sep 19;16:1662457. doi: 10.3389/fpls.2025.1662457 (PMC12491295; doi:10.3389/fpls.2025.1662457)
Supplement: Supplementary file 1 [file DataSheet1.zip › Supplementary Figure 1.pdf]

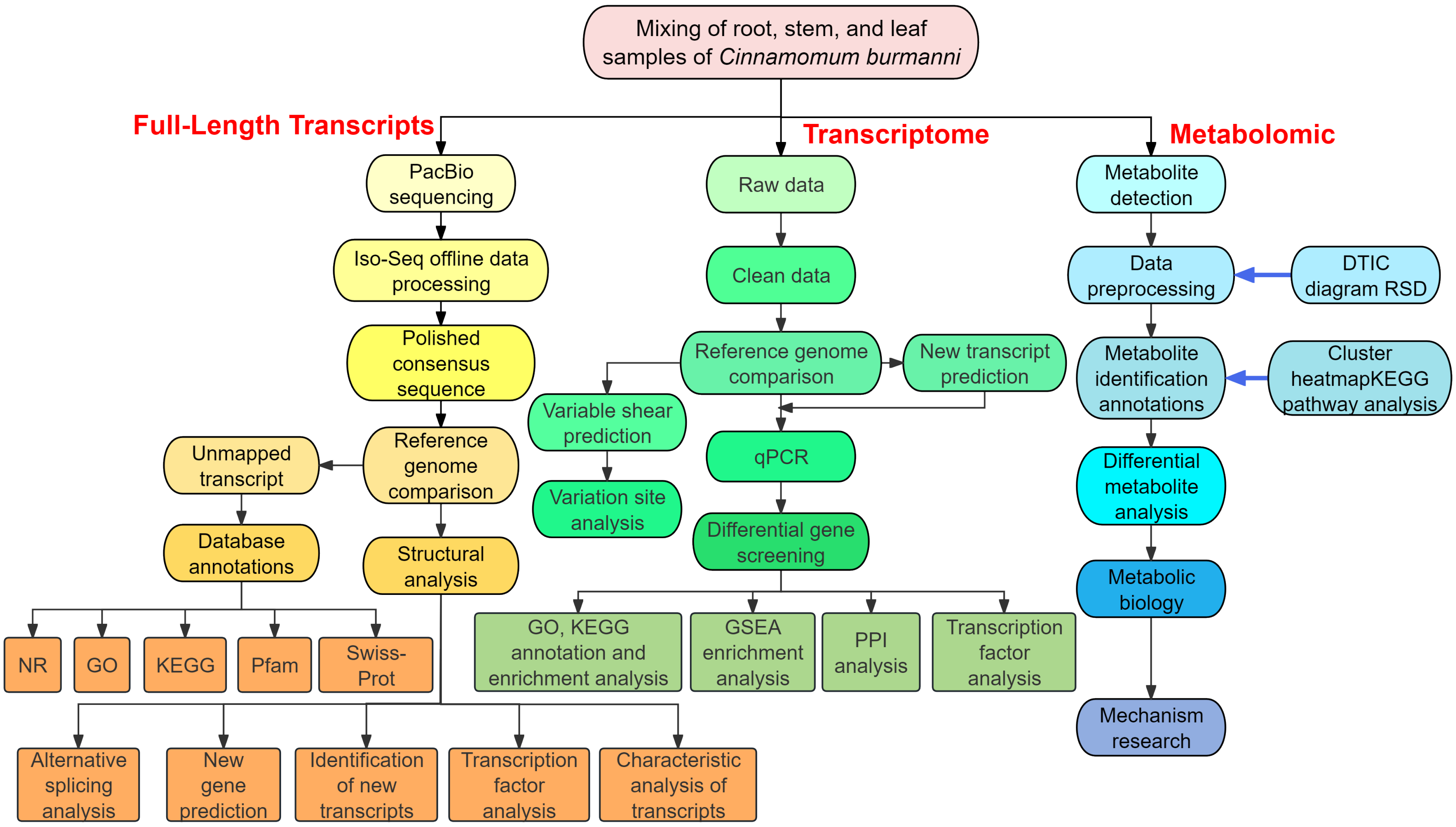

Supplementary Figure 1. Bioinformatics analysis workflow of full length transcriptome, transcriptome, and metabolome data in *Cinnamomum burmanni*
